# Transcription factor genetics and biology in predisposition to bone marrow failure and hematological malignancy

**DOI:** 10.3389/fonc.2023.1183318

**Published:** 2023-06-12

**Authors:** Jiarna R. Zerella, Claire C. Homan, Peer Arts, Anna L. Brown, Hamish S. Scott, Christopher N. Hahn

**Affiliations:** ^1^Adelaide Medical School, Faculty of Health and Medical Sciences, University of Adelaide, Adelaide, SA, Australia; ^2^Centre for Cancer Biology, SA Pathology and University of South Australia, Adelaide, SA, Australia; ^3^Department of Genetics and Molecular Pathology, SA Pathology, Adelaide, SA, Australia

**Keywords:** transcription factor, hematological malignancies (HM), bone marrow failure (BMF), germline, pathogenic variant

## Abstract

Transcription factors (TFs) play a critical role as key mediators of a multitude of developmental pathways, with highly regulated and tightly organized networks crucial for determining both the timing and pattern of tissue development. TFs can act as master regulators of both primitive and definitive hematopoiesis, tightly controlling the behavior of hematopoietic stem and progenitor cells (HSPCs). These networks control the functional regulation of HSPCs including self-renewal, proliferation, and differentiation dynamics, which are essential to normal hematopoiesis. Defining the key players and dynamics of these hematopoietic transcriptional networks is essential to understanding both normal hematopoiesis and how genetic aberrations in TFs and their networks can predispose to hematopoietic disease including bone marrow failure (BMF) and hematological malignancy (HM). Despite their multifaceted and complex involvement in hematological development, advances in genetic screening along with elegant multi-omics and model system studies are shedding light on how hematopoietic TFs interact and network to achieve normal cell fates and their role in disease etiology. This review focuses on TFs which predispose to BMF and HM, identifies potential novel candidate predisposing TF genes, and examines putative biological mechanisms leading to these phenotypes. A better understanding of the genetics and molecular biology of hematopoietic TFs, as well as identifying novel genes and genetic variants predisposing to BMF and HM, will accelerate the development of preventative strategies, improve clinical management and counseling, and help define targeted treatments for these diseases.

## Introduction

Over the last decade, the genomic revolution in combination with access to well curated clinical patient information and samples has enabled identification of rare germline syndromes of over-lapping and diverse clinical manifestations with different propensities for development of gene-specific bone marrow failure (BMF) and/or hematological malignancy (HM). Around 200 genes are currently included on high evidence germline targeted sequencing panels for HM predisposition, bleeding, and platelet disorders and BMF syndromes (1). The accruing identification of these predisposition genes helps to further inform leukemic biology and disease causation, allows for earlier diagnosis and genetic counseling for individuals who are at higher risk for the disease, permits the development of new and more effect treatments and, may improve our ability to develop preventative measures, targeting populations at higher risk of developing a HM.

Amongst the genes associated with HM and BMF, transcription factors (TFs) are commonly identified as mediators of hereditary predisposition and play a role in leukemogenesis by frequently exhibiting recurrent, somatically acquired chromosomal abnormalities and smaller point mutations and indels. The human genome contains over 1,800 genes that encode for TFs that display complex combinatorial interactions resulting in homeostatic transcriptional networks with positive and negative feedback loops to precisely regulate gene expression for cell fate trajectories and transitions, and cellular responses to environmental triggers (2, 3). Unironically, it is TFs that orchestrate gene expression regulation at each stage of hematopoietic development, including stem cell formation and maintenance, and lineage commitment and homeostasis (4, 5). Despite the identification of numerous master hematopoietic TFs as predisposition genes (inherited or *de novo*) (*RUNX1, CEBPA, GATA2, ETV6, PAX5, IKZF1*), it is likely that unrecognized predisposition genes and variants including in TFs will be discovered considering the complexity of the transcriptional network in normal hematopoietic stem cells (HSC), and the supporting bone marrow microenvironment. Stratifying BMF and/or HM TF predisposition genes currently relies on observed recurrence and functional evaluation, and excludes ontology, environmental factors, and somatic data (6). The prospective inclusion of these could provide a more comprehensive and accurate assessment of the genetic factors that contribute to BMF and/or HM development as well as for instance identification and inclusion of polygenic risk factors derived from multiple genetic variants of differing penetrance. Different types of variants in known BMF and HM predisposition genes display different pathogenic phenotypes and levels of penetrance (7).

Given the intricate interconnections among TFs and their multifaceted involvement in hematopoiesis, it is challenging to delineate the precise mechanism by which each TF aberration contributes to disease. It is suspected that TFs can contribute to disease using a wide range of mechanisms, including initiating the activation or repression of gene expression directly or through the recruitment of cofactors, epigenome changes, initiating new chromatin looping interactions between enhancers and target promoters, and altering the chromatin landscape through the repositioning of nucleosomes (8). This review will cover putative mechanisms leading to HM oncogenicity, including aberrations to hematopoietic TF networks, lymphoid and myeloid HM and BMF predisposition genes, and stress selection, the prospective inclusion of alternative data to TF stratification, as well as the potential of undiscovered predisposition TFs. Given other recent comprehensive reviews of BMF and/or HM predisposition (9–11) and the subjectivity of novel candidate inclusions to TF BMF and/or HM predisposition genes, this review will primarily focus on DNA binding predisposition TFs currently found in high evidence Genomics England PanelApp panels (Hematological malignancies cancer susceptibility [Version 3.3], Bleeding and Platelet Disorders [Version 1.16] Cytopenia’s and congenital anemias [Version 1.111]) (**Table 1**) and candidate TFs with an important role in normal hematopoiesis, reported leukemic association and/or oncogenic potential (**Tables 2**, **3**). As our knowledge of molecular TF biology expands, so will our ability to definitively establish HM disease diagnosis or reoccurrence, predict prognosis and response to therapy, tailor treatments, and ultimately implement prevention, management, surveillance, and treatment strategies (33–35).

**Table 1:** Known bone marrow failure and/or hematological malignancy predisposition transcription factors.

TF Groups	Gene symbol	Germline HM gene	Germline blood disorder gene	Canonical Transcript Coding size (aa)	TF Family	DNA binding motif	Human phenotype			Role in hematopoiesis (downstream differentiation)	Target genes
							OMIM		Mondo		ENCODE and ChEA Consensus TFs from ChIP-X
							Reported phenotype	Zygosity	Reported Phenotype #		
**Best characterized BMF/HM genes**	**RUNX1**	**Yes**	**Yes**	480	RUNX	TGTGG	AML Platelet disorder	AD	AML hereditary thrombocytopenia B-lymphoblastic leukemia/lymphoma with t(12;21)(p13.2;q22.1)	HSC, CMP, Megakaryocyte, Platelet, B-cell, T-cell	GATA2, GATA1, MAX, BRCA1, TCF3, CREB1, UBTF, TAF1, ZBTB7A
	**CEBPA**	**Yes**	**No**	358	bZIP	CCAAT T(T/G)NNGNAA(T/G)	AML	AD	AML	Granulocyte, Eosinophil, Monocyte, Neutrophil	ATF2, CREB1, CIN3A, CEBPD, NFYA, CREB1, NFYB, SIX5, GABPA
	**GATA2**	**Yes**	**Yes**	480	GATA	(A/T)GATA(A/G)	Emberger syndrome Immunodeficiency 21 AML MDS	AD	MDS/AML deafness-lymphedema-leukemia syndrome monocytopenia with susceptibility to infections	HSC, Mast cell, Erythrocyte, Basophil, Megakaryocyte	GATA1, RUNX1, SMAD4, TP63, NFE2L2, PPARG, UBTF, SPI1, RCOR1
**PAX5, MECOM, GATA1 and STAT3 BMF/HM genes**	**PAX5**	Yes	**No**	391	PAX	GGCTGAG	ALL	AD	ALL	Myeloid/lymphoid progenitor), B-cell	Not included in dataset
	**MECOM** **(MDS1/** **EVI1)**	Yes	Yes	1239	ZF	AAGA(C/T)AAGATAA	Radioulnar synostosis with amegakaryoctiyc thrombocytopenia	AD	AML MECOM-associated syndrome radioulnar synostosis with amegakaryyocytic thrombocytopenia	HSC	SPI1, RUNX1, GATA1, GATA2, FLI1, ZNF384, FOS, RCOR1, TCF3
	**STAT3**	Yes	Yes	770	STAT	TTCC(C/G)GGAA	Autoimmune disease Hyper-IgE recurrent infection syndrome	AD AR	STAT3-related early-onset multisystem autoimmune disease hyper-IgE recurrent infection syndrome 1	HSC HPC	AR, SMAD4, SUZ12, REST, TCF3, SOX2, POU5F1, NFE2L2, NANOG
	**GATA1**	Yes	Yes	413	GATA	(A/T)GATA(A/G)	Anemia Hemolytic anemia Leukemia Thrombocytopenia with beta-thalassemia Thrombocytopenia with/without dyserythropoeitic anemia	XLR	GATA1-Related X-Linked Cytopenia AML thrombocytopenia transient myeloproliferative syndrome	Eosinophil, Platelet, RBC Basophil, Mast-cell, Erythrocytes, Dendritic cell, Megakaryocyte, Macrophage, MPC	GATA2, RUNX1, SMAD4, AR, ZBTB7A, SPI1, UBTF, ESR1, STAT3
**ETS BMF/HM** **genes**	**ETV6** **(TEL)**	**Yes**	**Yes**	452	ETS	(G/C)CGGAAGT(G/A)	AML Thrombocytopenia	AD	Otosclerosis	HSC Megakaryocyte	Not included in dataset
	**FLI1**	**No**	**Yes**	452	ETS	AGGAA(G/A)	Bleeding disorder	AR AD	Nephronophthisis, Thrombocytopenia, Otosclerosis, paragangliomas, anencephaly, odontochondrodysplasia, schizophrenia, trigonocephaly, thrombocythemia, chondrocalcinosis	Platelet	FLI1, ELF1, SPI1, TAF1, ATF2, RUNX1, BRCA1, CREB1, NRF1
**IKAROS** **BMF/HM** **genes**	**IKZF1**	**Yes**	**Yes**	519	IKAROS	GGAAA	Immunodeficiency	AD	No Phenotypes reported	T-cell, HSC, CLP	ZKSCAN1, VDR, RCOR1, REST, SUZ12, NFE2L2, SPI1, UBTF
	**IKZF3**	**Yes**	**Yes**	509	IKAROS	(T/G)GGAA	Immunodeficiency	AD	No Phenotypes reported	B-cell, T-cell	Not included in data set
	**IKZF5**	**No**	**Yes**	419	IKAROS	GNNTGTNG	Thrombocytopenia	AD	No Phenotypes reported	HSC	Not included in data set

Germline HM and/or blood disorder predisposition genes, found in high evidence Genomics England PanelApp panels: Hematological malignancies cancer susceptibility [Version 3.3] and/or Bleeding and Platelet Disorders [Version 1.16] as well as Cytopenias and congenital anemias [Version 1.111]. Known interacting partners were collated through STRING (12), target genes through ChEA from Chip-X (13), and roles in hematopoiesis literature (14–24). Autosomal dominant, AD; Autosomal recessive, AR.

**Table 2:** Oncogenic potential of hematopoietic-related TFs.

	TF gene symbol	Functional domain	MGI Mouse BMF/HM phenotype	Embryonic lethality			Constraint				COSMIC no. of LOFs	Malignant fusions		Biallelic Variants Observed	Known CHIP genes
				Het mice	Hom mice	Human	LOEUF	pLI	Domain	Average constraint		Somatic	HM specific		
**Known BMF/HM TFs**	**RUNX1**	RUNT TAD	Yes	No	Yes	NA/ Miscarriage observed	0.44	0.65	RUNT	0.48, intolerant	819 (23.9%)	AML1/MDS1, AML1/ETO, AML1/MDS1/EAI1, AML1/FOG2, RUNX1/YTHDF2, RUNX1/SH3D19, RUNX1/ZNF687	AML1-ETO AML1-FOG2 AML-TEL	Yes	Yes (rare)
	**CEBPA**	TAD1 TAD2 DBD Zip	Yes	No	Die ~4-10 h after birth	NA	1.18	0.55	Zip	0.5032, intolerant	711 (43.1%)	No somatic lesions found	No HM specific malignant fusions found	Yes	No
	**GATA2**	TAL1 TAL2 ZF 1 ZF 2	Yes	No	Yes	NA/ Miscarriage observed	0.29	0.98	ZF 1 ZF 2	0.13, highly intolerant 0.149, highly intolerant	63 (6.67%)	inv(3)(q21q26)	inv(3)(q21q26)	No (rare)	No
	**PAX5**	OP, HD TA ID	Yes	No	Yes	NA	0.17	1	Paired domain	0.23, intolerant	80 (4.3%)	No somatic lesions found	PAX5/NOL4L	Yes	No
	**MECOM/EVI1**	ZF (x10) AD	Yes	No	Yes	Yes	0.14	1	ZF, ZF, ZF	0.24, intolerant 0.28, intolerant 0.08, highly intolerant	115 (8.69%)	EVI1/RBPH1	EVI1/GR6	No	No
	**STAT3**	NTD, CC, DBD, LD, SH2, TAD	Yes	No	Yes	NA/ Miscarriage observed	0.10	1	NTD, CC, DBD, LD, SH2, TAD	0.19, intolerant	56 (3.67%)	No somatic lesions found	No HM specific malignant fusions found	No	Yes
	**GATA1**	AD ZF 1 ZF 2	Yes	No	Yes	Yes (Males)/ Miscarriage observed	0.32	0.95	ZF 1, ZF 2	0.18, intolerant 0.17, highly intolerant	269 (34.1%)	No somatic lesions found	MYB-GATA1	No	No
	**ETV6** **(TEL)**	HLH ETS	Yes	No	Yes	NA/ Miscarriage observed	0.32	0.97	ETS	0.173, highly tolerant	126 (7.3%)	ETV6/PDGFRB, ETV6/MDS2, ETV6/NTRK3,	ETV6/AML1, ETV6/JAK2, ETV6/RUNX1, ETV6/MN1, ETV6/ACS2, ETV6/ABL2, ETV6/ARNT, ETV6/BTL, ETV6/PER1	Yes	No
	**FLI1**	ETS	Yes	No	No	NA	0.28	0.99	ETS	0.0953, highly intolerant	22 (1.97%)	FLI1/EWS	No HM specific malignant fusions found	Yes	No
	**IKZF1**	ZF (x6)	Yes	No	Yes	NA	0.16	1	ZF	0.1086, highly intolerant	80 (3.41%)	No somatic lesions found	IKAROS/BCL6	No	No
	**IKZF3**	ZF (x6)	Yes	No	No	NA	0.29	0.98	ZF	0.31, intolerant	29 (2.80%)	No somatic lesions found	No HM specific malignant fusions found	No	No
	**IKZF5**	ZF (x5)	Yes	No	No	NA	0.46	0.78	ZF	No data available	32 (13.56%)	No somatic lesions found	No HM specific malignant fusions found	No	No
**Strong candidate BMF/HM TFs**	**GATA3**	TAL1 TAL2 ZF 1 ZF 2	Yes	No	Yes	Yes	0.39	0.9	ZF 1, ZF 2	0.12, highly intolerant, 0.31, intolerant	784 (43.1%)	No somatic lesions found	No HM specific malignant fusions found	No	No
	**SPI1** **(PU.1)**	AD PEST ETS	Yes	No	Yes	NA	0.24	0.98	ETS	0.1261, highly intolerant	6 (1.86%)	No somatic lesions found	No HM specific malignant fusions found	No	No
	**ERG**	PNT ETS	Yes	No	Yes	NA	0.33	0.96	ETS	0.2526, intolerant	66 (3.42%)	ERG1, ERG2, ERG/TMPRSS2, ERG/EWS, ERG/FUS	ERG/FUS	No	No

Oncogenic potential of known BMF and/or HM predisposition TFs, as well as strong candidate BMF and/or HM predisposition TFs mapped using mouse phenotypes, constraint scores, variants observed and malignant fusions. Mouse phenotypes collated through Mouse MGI (25–27), and mousephenotype.org (28), constraint scoring (LOEUF, pLI and No. of LOF) extracted from gnomAD (29), domain constraint calculated from MetaDome (30), and somatic mutations obtained from COSMIC (31).

NA, not applicable.

**Table 3:** Candidate inclusions to BMF/HM predisposition gene lists.

Gene symbol	Canonical Transcript Coding size (aa)	TF Family	Human phenotype		Constraint				gnomAD No. of LOF (different/ total)	COSMIC		Murine embryonic lethality		Malignant Fusions	
			Phenotype	Zygosity	LOEUF	pLI score	Domain	Average domain constraint		point mutations in Hematopoietic tissue/total samples (%)	No. of LOF	Het	Hom	Somatic	HM specific
**ETV2**	342	ETS	None reported	N/A	1.42	0	ETS	0.5563, SI	28/121	7/5924 (0.12%)	10/155 (6.45%)	No	Yes	None observed	None observed
**ELF1**	501	ETS	None reported	N/A	0.58	0.01	ETS	0.4664, I	17/19	53/8011 (0.66%)	39/659 (4.7%)	No	No	None observed	None observed
**FEV**	238	ETS	None reported	N/A	1.32	0.01	ETS	0.3402, I	5/7	4/5924 (0.07%)	4/99 (4.0%)	No	No	FEV/EWS	None observed
**ETS1**	485	ETS	None reported	N/A	0.39	0.78	ETS	0.221, I	7/7	136/6148 (2.21%)	32/1086 (2.9%)	No	No	None observed	None observed
**ETS2**	469	ETS	None reported	N/A	0.2	1	ETS	0.23, I	5/8	22/5924 (0.37%)	17/401 (4.2%)	No	Yes	None observed	None observed
**IKZF2**	526	IKAROS	None reported	N/A	0.29	0.99	ZF	0.22, I	6/157	71/6587 (1.08%)	35/1124 (3.11%)	No	No	None observed	None observed
**IKZF4**	585	IKAROS	None reported	N/A	0.38	0.9	ZF	0.11, HI	9/11	20/5924 (0.34%)	13/423 (3.07%)	No	No	None observed	None observed
**GFI1B**	330	Proto-oncogene	Bleeding disorder, platelet-type, 17*	AD, AR	0.86	0	ZF ZF ZF ZF	0.53, SI 0.51, I 0.34, I 0.7241, N	16/52	28/5924 (0.47%)	11/500 (2.2%)	No	Yes	None observed	None observed
**TAL1** **(SCL)**	331	bHLH	Leukemia, T-cell acute lymphocytic	Somatic	0.7	0.57	BHLH	0.35, I	10/47	19/6120 (0.31%)	34/398 (8.54%)	No	Yes	None observed	None observed
**LYL1**	280	bHLH	Leukemia, T-cell acute lymphoblastoid	Not recorded	0.61	0.77	BHLH	0.50, I	3/4	7/5924 (0.12%)	8/127 (6.3%)	No	No	None observed	None observed
**LMO2**	227	bHLH	Leukemia, acute T-cell	Not recorded	1.028	0.16	LIM 1 LIM 2	0.54, SI 0.391, I	9/13	31/6037 (0.51%)	6/358 (1.68%)	No	Yes	None observed	None observed
**MEIS1**	390	Homeo-box	None reported	N/A	0.14	1	MEIS Homeo-box	0.19, I 0.045, HI	2/2	102/5924 (1.72%)	18/1094 (1.65%)	No	Yes	None observed	None observed
**TCF12**	706	bHLH	Craniosynostosis 3, Hypogonadotropic hypogonadism 26 with/without anosmia	AD, AR	0.37	0.68	Helix-loop-Helix DNA BD	0.2837, I	24/29	113/6172 (1.83%)	67/1550 (4.3%)	No	Postnatal lethality within two weeks	TCF12/NR4A3	None observed
**GFI1**	422	Proto-oncogene	Neutropenia, severe congenital 2*	AD	0.56	0.25	ZF 1 ZF 2 ZF 3	0.2678, I 0.6286, SI 0.6491, SI	11/13	15/6073 (0.25%)	20/308 (6.5%)	No	No	None observed	None observed
**FOS**	380	FOS	None reported	N/A	0.65	0.26	bZIP TF	0.3376, I	6/6	8/5924 (0.14%)	16/211 (7.6%)	No	No	None observed	None observed
**HOXA9**	272	Homeo-box	None reported	N/A	1.62	0	Homeo-box Domain	0.4315, I	13/41	14/6233 (0.22%)	5 (2.33%)	No	No	None observed	HOXA9/NUP98, HOXA9/MSI2
**FOSB**	338	FOS	None reported	N/A	0.3	0.98	bZIP TF	0.16, HI	6/6	9/5924 (0.15%)	11 (3.45%)	No	No	None observed	None observed
**FOXC2**	501	Forkhead box	Lymphedema-distichiasis syndrome with renal disease and diabetes mellitus	AD	0.79	0.13	Forkhead Domain	0.21, I	6/7	3/5924 (0.05%)	5 (1.66%)	No	Yes	None observed	None observed
**EKLF (KLF1)**	362	KLF	[Hereditary persistence of fetal hemoglobin], Dyserythropoietic anemia, congenital, type IV, Blood group–Lutheran inhibitor, - Ineffective erythropoiesis	AD	1.13	0	ZF	0.83, N	16/34	5/5924 (0.1%)	2 (1%)	No	Yes	None observed	None observed
**NFKB1**	969	NF-kappaB	Immunodeficiency, common variable, 12,	AD	0.16	1	Ankyrin repeat 1, Ankyrin Repeat 2, Death Domain	0.52, I 0.57, SI 0.63, SI	10/82	67/6967 (0.96%)	18 (1.98%)	No	No	None observed	

*GFI1 (S36N) polymorphism is associated with incidence and/or prognosis of AML, MDS and multiple myeloma (32). Candidate inclusions to BMF/HM predisposition gene lists, not found in high evidence Genomics England PanelApp panels: Hematological malignancies cancer susceptibility [Version 3.3] and/or Bleeding and Platelet Disorders [Version 1.16] + Cytopenias and congenital anemias [Version 1.111]. TFs are isolated by involvement in hematopoiesis, oncogenic potential, and previous disease association. Human phenotype is collated through OMIM. Slightly intolerant, SI; intolerant, I; highly intolerant, HI; neutral, N; loss-of-function observed over expected upper bound fraction, LOUEF.

NA, not applicable.

## Oncogenic TF networks in hematopoiesis

Hematopoiesis is a complex process in which multiple regulatory TF pathways converge to orchestrate tissue formation, cell movement and cell fate decisions including lineage specification and differentiation. Analysis of genome wide binding patterns identified ten key TF regulators of hematopoietic stem/progenitor cells (HSPC) (i.e., TAL1, LYL1, LMO2, GATA2, RUNX1, MEIS1, PU.1, ERG, FLI1 and GFI1B) (36). The transcriptional role of each TF in HSPCs has been somewhat demonstrated, with combinatorial interaction studies suggesting highly cooperative control of transcription by a core set of seven TFs (i.e., FLI1, ERG, GATA2, RUNX1, SCL/TAL1, LYL1, LMO2) (36). During hematopoiesis, TFs form densely interconnected spatially and temporally regulated networks which either engage regions through cognate motifs or bind indirectly *via* the formation of protein-protein interactions (37). If a single player within the network is mutated, it can lead to TF dysregulation of interacting partners or target genes, impaired differentiation, fuel immature cell population growth and/or trigger inappropriate transcriptional programs; all of which may initiate malignancy. This highlights that small genetic changes in TFs can have a wide-ranging impact, affecting many of the components of the transcriptional network of which they are a part (38).

**Figure 1:**
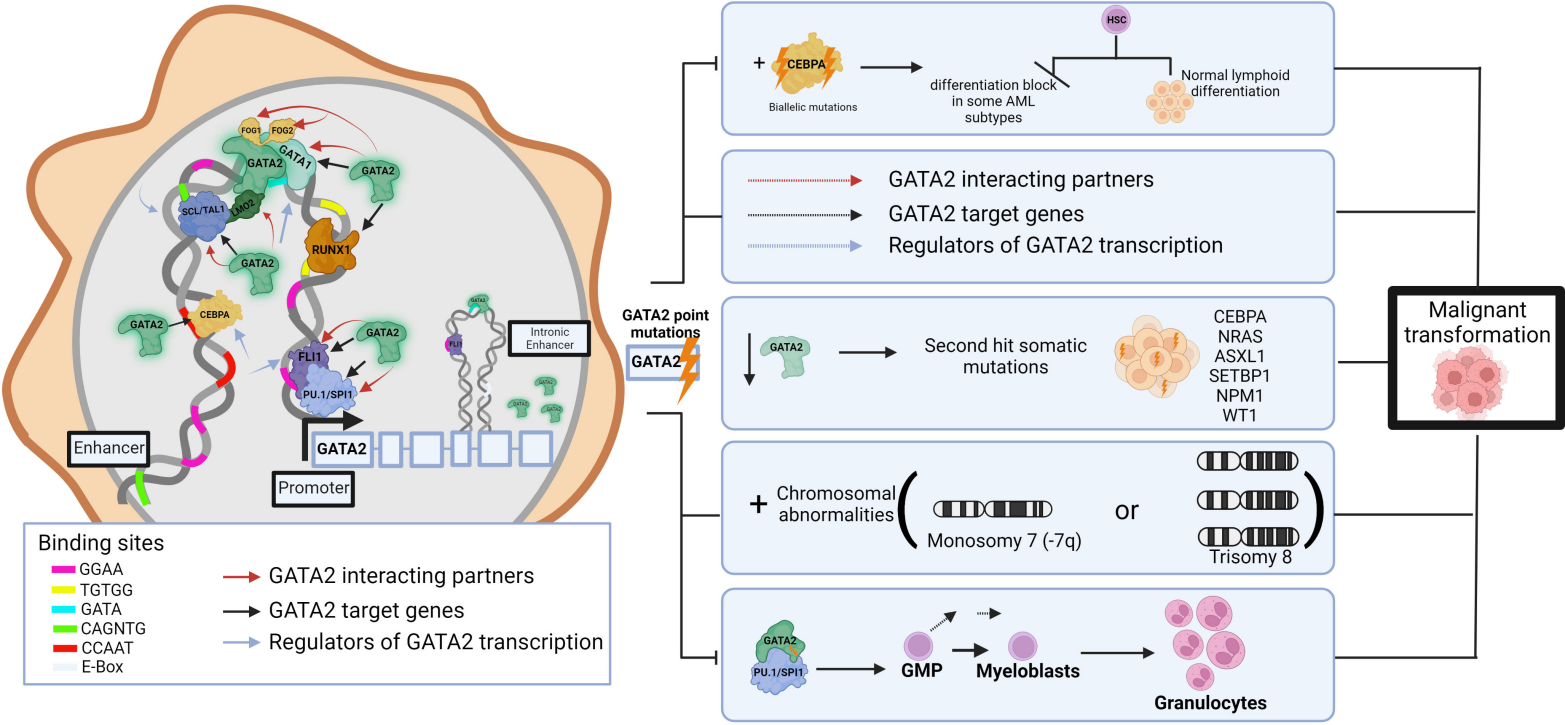
Impact of germline pathogenic variants in GATA2 on hematopoietic network and leukemic transformation. Schematic of the role of GATA2 interactions in transcriptional networks in normal hematopoiesis including interacting partners, target genes and upstream regulators. GATA2, a common target of aberration in leukemias, leads to inappropriate transcriptional programs resulting in TF dysregulation, impaired differentiation, and subsequent expansion of immature cell populations to initiate malignancy. While based on published data, the figure is somewhat hypothetical as each hematopoietic TF gene regulatory region is bound by a different combination, clustering, and arrangement of these TFs. Pathogenic germline variants (lightning bolt) may lead to disrupted DNA binding and/or interactions with other TFs (dashed arrows) resulting in a range of impacts on downstream target genes that may include disruptions to additive, synergistic and/or inhibitory transcriptional events. This image was created using BioRender.com.

The TF network regulates multiple aspects of normal HSPC function and thus components of these transcriptional networks are a common target of aberration in leukemias, resulting in selection of cells with a leukemic ‘stem cell’ signature (39). For example, dysregulation of GATA2, a master regulator of hematopoiesis, causes hematologic pathologies. Around 80% of germline GATA2 carriers develop a myeloid malignancy before the age of 40 years (40), and it is shown that the progression to malignancy can occur through multi-step secondary events targeting various hematopoietic pathways. These include (but are not limited to) acquired biallelic mutations in CEBPA (41, 42) which lead to differentiation blocks in some AML subtypes (38), NRAS, ASXL1, SETBP1, NPM1, and WT1 secondary mutations (43), chromosomal abnormalities (monosomy 7 or trisomy 8) (44)), disrupting the regeneration abilities of enhancers (45), HSC exhaustion following repeated infections, and changes in DNA binding affinities that result in disruptions to interaction partners/target genes. (**Figure 1**) For example, the GATA2 p.T354M missense variant may contribute to the leukemogenic process in two ways, by partial loss of GATA2 transactivation activity and simultaneous increased affinity to PU.1 (46), thereby potentially interfering with differentiation and driving cells towards granulocytic disease (7). Further, GATA2 p.R396Q leads to complete loss of DNA binding and transactivation, and inability to maintain the undifferentiated characteristics of HSCs (47). Single-cell transcriptomics identified 196 TF regulatory networks in AML patient cells and used this data to identify specific TF sub-networks that play a key role in determining differentiation trajectories in hematopoiesis (37, 48). For example, FLI11/ERG constitute a sub-network that promotes endothelial cell fate, whilst another RUNX1/GATA2 sub-network has been implicated in the promotion of cells to a hematopoietic cell fate (49). Cell-type transitions such as these are under tight transcriptional control, and loss of this control by any TF aberration can also lead to proliferation and leukemic transformation (38). Interestingly, analysis of heptad expression identified RUNX1, FLI1, LMO2, GATA2, ERG and LYL1 levels were significantly higher in groups of poorer overall survival, suggesting that high expression levels of the heptad contribute to the relative immaturity or stemness of the AML transcriptome (4, 39).

Evidently, networks can be disrupted *via* multiple pathways and result in dysregulation of the transcriptional network, a potential reason for phenotypic diversity observed in germline carriers. Given this knowledge it is intriguing to hypothesize that germline variants in genes of the core hematopoietic TF network may be implicated in leukemogenesis. Some are currently included in high evidence Genomics England PanelApp panels for HM and/or BMF susceptibility (i.e., *GATA2, RUNX1, FLI1, GFI1B*), while the significance of others (i.e., *ERG, SPI1* (encoding PU.1 protein)) remain to be determined.

## Gene-specific mechanisms of oncogenicity

While TFs that predispose to BMF and/or HM may reside and act in common networks, no two TFs predispose to exactly the same phenotypes with the same hematopoietic disorder(s) incidence rate, average age of onset or propensity to solid cancers. Modern efforts to characterize genes for germline predisposition to HM has yielded a much larger genetic susceptibility than previously thought (50). Observed germline occurrence has so far allowed for the identification of predisposition germline mutations encoding master hematopoietic TFs such as RUNX1, CEBPA, GATA2, ETV6, PAX5 and IKZF1 (IKAROS). The majority of these segregate as autosomal dominant in nature. In the following section we review the known predisposition BMF and/or HM TF genes.

### Best characterized TF BMF and/or HM predisposition genes: RUNX1, GATA2 and CEBPA

**Figure 2:**
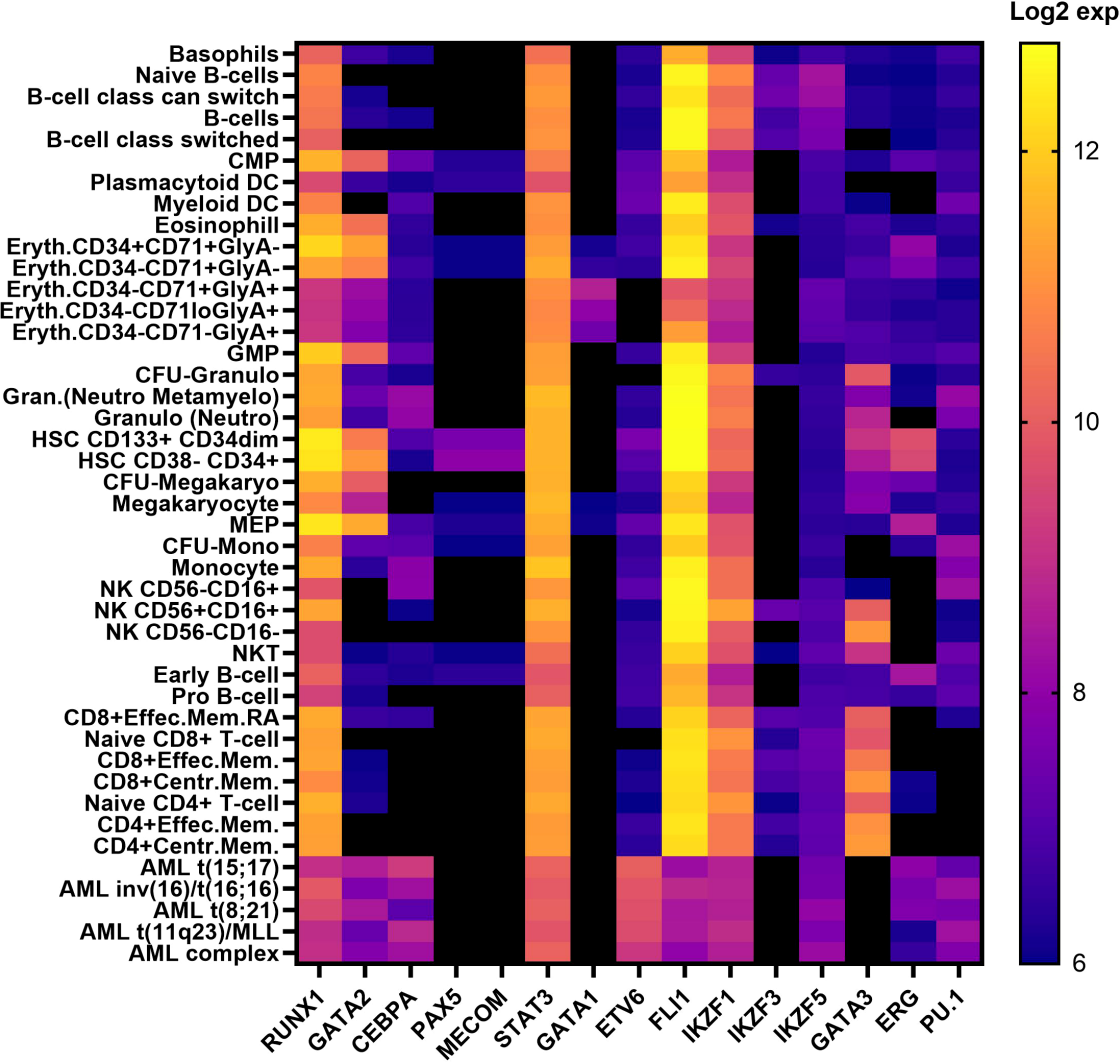
Transcription factor expression in hematopoietic cells in human hematopoiesis Vs AML. Heatmap correlating the expression levels of TFs in hematopoietic cells in human hematopoiesis with AML. The mRNA expression levels of microarray data (log2) in the ‘Normal human hematopoiesis (DMAP)’ and ‘normal hematopoiesis with AML’ datasets from BloodSpot were used, where the probe with the overall highest intensity was selected and each quadruplicate/triplicate/duplicate averaged. TFs included those selected in **Table 1** (56). common myeloid progenitor, CMP; dendritic cell, DC; Erythroid, Eryth; granulocyte/monocyte progenitor, GMP;CFU; colony forming unit, granulocyte, Gran; megakaryocyte/erythroid progenitor, MEP; mature natural killer, NK; natural killer T cell, NKT; memory. mem.

*RUNX1, GATA2 and CEBPA* are archetypal HM predisposition genes. First reported well over a decade ago, germline RUNX1 (51), GATA2 (46) and CEBPA (52) are the best characterized TF genes linked to HM predisposition. Unlike preleukemic syndromic features often associated with RUNX1 and GATA2, CEBPA variants predispose solely to AML without any recognized preleukemic phenotypes (53, 54). These three predisposition genes harbor point mutations and small indels (both somatic and inherited), resulting in leukemia, in addition to partial or whole gene deletions being observed in RUNX1 and GATA2-driven HM. RUNX1 also presents with over 70 translocation fusion partnerships in patients with HM (55). RUNX1, GATA2 and CEBPA all reside in similar hematopoietic networks, impacting common and different regulatory pathways, however each play unique roles during hematopoiesis and in cell fate decisions, despite the expression levels of RUNX1 in hematopoietic cells presenting significantly higher than both GATA2 and CEBPA (**Figure 2**) (14–19, 57). RUNX1 binds to the TGTGGT DNA-binding motif as a heterodimer with core binding factor beta (CBFβ) and can activate or repress gene expression depending on its cellular context and in response to extracellular signals from the microenvironment. This includes regulation of chromatin accessibility and its role in recruiting and enacting transcriptional programs with other transcriptional co-factors (58–60). CEBPA and GATA2 are predominantly myeloid transcription factors which bind to CCAAT and GATA motifs (**Table 1**), respectively, but have distinct roles in regulating myeloid differentiation. (**Table 1**) (14, 15, 18–20).

Pathogenic germline variants contributing to RUNX1/GATA2-driven disease are largely premature terminations throughout the coding region or missense variants, clustering in the DNA binding runt homology domain (RHD) of RUNX1 and C-terminus (zinc finger (ZF) 2 domain) of GATA2, predominantly resulting in haploinsufficiency (53, 54). Interestingly, no pathogenic germline GATA2 single nucleotide variants have been described within the N-terminus, except for two rare variants, p. T117= (c.351C>G) (61–63) and p.A286V (c.857C>T) (64, 65), both of which generate strong cryptic splice sites and are better classified as truncating splice variants leading to nonsense mediated decay (53). There are also very rare individual cases of variants in ZF1 (p.H313Y, p.L315P, p.A318T) (53), which intriguingly is the most common site of somatic mutations (66). Strikingly, there is a clear separation of GATA2 germline (ZF2) and somatic (ZF1) missense variants in HM, suggesting different mechanisms of leukemogenesis in germline GATA2 HM. In contrast, distinction of germline RUNX1 and sporadic HM variants is challenging as identical variants are observed in both. Germline deletions of RUNX1, only affecting the RUNX1c isoform support this as being the predominant oncogenic isoform (9).

Germline CEBPA mutations are most commonly frameshift, protein-truncating variants at the N-terminus with rare families harboring C-terminal in frame insertions or deletions (54, 67). The N-terminal premature termination prevents generation of the full-length 42 kDa protein but preserves translation of the smaller 30 kDa isoform (68). These mutations display a high degree of penetrance for AML (90%) compared to C-terminal in-frame indels that display lower penetrance (50%) (52, 54, 68). Individuals with a germline CEBPA N-terminal mutation at AML diagnosis, often acquire C-terminal CEBPA mutations within the bZIP region (predominantly missense or in-frame indels), highlighting the synergistic effect of these lesions and their selection during clonal expansion (69). In <10% of cases however, homozygous single N- and C- terminal mutations have been reported, arising from copy neutral loss of heterozygosity (69). Like RUNX1, CEBPA germline and somatic variants may look identical, and a germline tissue sample or familial segregation with disease is necessary for confirmation of germline origin.

The dysregulation of RUNX1, GATA2 and CEBPA activity underlies hematologic pathologies, yet the biological mechanisms that establish and maintain its contextually distinct expression patterns are versatile. GATA2 and RUNX1 have been shown to function as both “pioneer” and “master” regulatory TFs in hematopoiesis, opening chromatin for easy access by other TFs and recruiting other TFs to enhance downstream effectors and signaling pathways (70). Their direct associations with other important hematopoietic TFs such as with each other, TAL1 and PU.1 (**Table 1**) (12, 13), may highlight a mechanism by which missense variants or reduced levels of the wildtype (haploinsufficiency) protein disrupt the stoichiometry required for normal hematopoietic expansion and differentiation processes leading to microenvironments that are conducive to development of cytopenia’s or initiation, maintenance and/or progression of malignancy. Complete deletion of either the RUNX1 or GATA2 locus suggests haploinsufficiency as the mechanism of predisposition; however, not all variants result in complete LOF of one allele. For example, GATA2 p.T354M and RUNX1 p.R204Q missense pathogenic variants display not only partial loss of transactivation activity, but also act in a dominant negative manner (46, 71). It is not clear whether the dominant negative action further decreases the total cellular activity levels in these partial LOF variants to approach that of the complete LOF situation.

Amidst germline RUNX1, GATA2 and CEBPA variants setting the basis for autosomal dominant predisposition to leukemogenesis, the considerable clinical heterogeneity in disease progression (even within families) suggests they are not transformation-sufficient, and that acquired secondary mutations are required for initiation and maintenance of malignancy (72) and may dictate the type of malignancy and its response to therapies. Ironically, the number of LOF mutations found in Catalog of Somatic Mutations in Cancer [COSMIC (73)] for RUNX1 and CEBPA surpass other hematopoietic TFs by hundreds (**Table 2**), coinciding to the hypothesis that the acquisition of somatic mutations in these oncogenic genes strongly drives leukemic transformation. The selective advantage of clones is often shaped by the acquisition of new aberrations in distinct genes. For example, STAG2 mutations act as a driver of clonal hematopoiesis with limited leukemic potential in GATA2 deficiency patients, but are rarely seen outside of this context, while SETBP1, RUNX1 and RAS pathway mutations along with monosomy 7 are associated with leukemic transformation (74). Molecular analysis of GATA2 and RUNX1 deficiency by germline predisposition found that the most frequent cytogenetic abnormalities for GATA2 involved monosomy 7 or trisomy 8, and the aggregation of second hit somatic mutations were unique to each gene with GATA2, targeting most frequently ASXL1, NRAS/KRAS, WT1, STAG2, and SETBP1, whereas in RUNX1, most frequently biallelic RUNX1 variants, followed PHF6, BCOR, WT1, and TET2, leading to leukemic transformation (54, 72, 74). Acquisition of somatic RUNX1 variants appears to be a late event associated with disease transformation, with clonal hematopoiesis (driven by BCOR, TET2) observed in pre-leukemic carriers.

**Figure 3:**
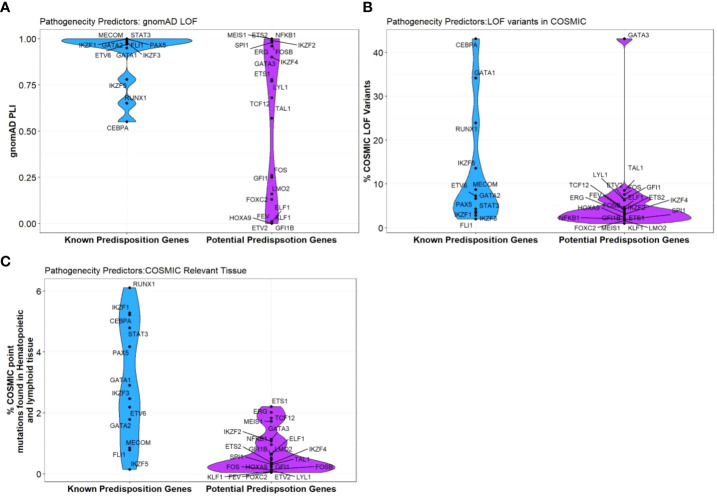
Pathogenicity predictors for TFs implicated in predisposition to BMF and HM. Comparison of pathogenicity predictor score for known predisposition TFs and potential TF genes isolated from **Table 1**. **(A)** The Probability of being loss-of-function (LOF) intolerant (pLI) score of each gene was collated using the gnomAD database (version 2.1.1). The pLI score reflects the tolerance of a given gene to the LOF based on the number of protein-truncating variants referenced in control databases weighted by the size of the gene and the sequencing coverage. The pLI score ranges from 0-1, where higher the score, the higher the intolerance of the gene. **(B)** Percentage of gene LOF variants in COSMIC database. The total number of somatic LOF variants within a particular gene were calculated by totaling positive mutation data for the selected gene. Variants called ‘LOF’ included nonsense substitutions, frameshift insertions and frameshift deletions. Percentage calculated using LOF variants over the total number of unique samples of each gene. **(C)** Percentage of point mutations observed in hematopoietic and lymphoid tissues. The distribution of mutations across the primary hematopoietic and lymphoid tissues curated by COSMIC were collated. The percentages were calculated by totaling the number of point mutations of each gene, over the total samples tested.

Curiously, RUNX1, GATA2 and CEBPA display loss-of-function (LOF) intolerant (pLI), LOF observed/expected upper bound fraction (LOEUF) and domain constraint (metadome) scores no higher than many other TFs associated with hematopoiesis, (**Figure 3**, **Table 2**) (29, 30) suggesting that it is likely that other TF genes remain to be identified in BMF and/or HM predisposition.

### The ETS family of BMF/HM predisposition TFs

ETS TFs, are a family of TFs which share a winged helix-turn-helix DNA binding domain (ETS domain), which recognize the DNA sequence GGAA/T. ETS TFs (e.g. ETS1, ETS2, ELF1, ERG, ETV2, ETV6, FEV, FLI, PU.1) are involved in the regulation of a variety of genes in hematopoiesis; however despite this involvement, only ETV6 (also known as TEL) and FLI1 have been included on germline targeted sequencing panels for bleeding and platelet disorders and HM predisposition (1). ETV6 primarily functions as a transcriptional repressor, targeting a wide spectrum of genes, many of which are highly regulated during hematopoiesis, whilst FLI1 plays an essential role in embryogenesis, vascular development and megakaryopoiesis (75–77). Interestingly, germline pathogenic variants in both TFs co-segregate with autosomal dominant bleeding disorders (i.e., mild thrombocytopenia), although ETV6 also presents a HM risk in ~30% of carriers while FLI1 is most commonly autosomal recessive (78–81). As a consequence of the important function of other ETS TFs (e.g. ETS1, ETS2, ELF1, ERG, ETV2, FEV, PU.1) in hematopoiesis (82), including the role of ERG in definitive hematopoiesis, adult HSC function and platelet maintenance (83), and the role of PU.1 in positive regulation of genes in the macrophage, granulocyte, dendritic-cell and B-cell lineages, (**Table 4**) (84) it is probable that germline genetic changes involving these TFs will also be found to predispose or contribute to aberrant hematopoiesis including cytopenia’s and/or HM (85). As with the intron 4 enhancer variants in GATA2 (86), causal genetic variants may well be in gene elements such as promoters, enhancers and/or suppressors that are used or activated in a cell-specific manner at particular stages of hematopoietic lineage development and maturation. In keeping with this, using Genome-wide association studies (GWAS) for some cancers such as breast cancer, common genetic variants have been identified in intergenic and intronic regions and shown to impact on important regulatory regions such as promoters and enhancers of known and novel genes important for the cancer initiation or development as well as multi-exonic non-coding RNA (mencRNA) genes (87, 88). It is conceivable and likely that rare variants may similarly exist that have even greater impact on cancer including HM predisposition, and such regulatory regions are not screened in WES, not included in panels, and not interrogated or not interpretable in WGS. To date, detection of germline variants for most hematopoietic and HM genes including TF genes have been confined to the coding regions with exceptions such as GATA2 intron 4 enhancer (86), ANKRD26 5’UTR (89) and TERT promoter (90).

**Table 4:** Strong candidate inclusions to BMF/HM predisposition gene lists.

TF gene symbol	TF Family	DNA Binding Motif	Reported Human phenotype (Hematological)		MGI Mouse Phenotype (Hematological)	Role in hematopoiesis (downstream differentiation)	Known Interacting TF partners (STRING)	Target genes (TFs)
			OMIM	MONDO	Phenotypes			ENCODE and ChEA Consensus TFs from ChIP-X
**GATA3**	GATA	WGATAR	N/A	N/A	Abnormal T cell physiology and differentiation Abnormal definitive hematopoiesis Abnormal erythrocyte morphology	T Cells, HSC, NK cells, B cells, CLP	TAL1, IL5 IL33, IL33, STAT6, TBX21, FOXP3, IL13, SMAD3, ESR1, FOXA1	GATA1, SALL4, KLF4, MYC, E2F1, REST, TRIM28, SPI1, STAT3
**SPI1** **(PU.1)**	ETS	GGAA	Decreased/absent circulating B cells Impaired B-cell development Lymphopenia Neutropenia	N/A	Transient neutropenia Abnormal granulocyte, neutrophil, macrophage, blood cell and B/T cell differentiation Increased hematopoietic stem cell proliferation/abnormal definitive hematopoiesis Abnormal common myeloid progenitor cell morphology Abnormal erythropoiesis, leukopoiesis	Monocyte, B cells, GMP, CLP, CMP, Granulocyte	IRF4, GATA1, CSF3R, EP300, JUN, TBP, GATA2, IRF8, CEBPE, CEBPA	SPI1, ELF1, FLI1, GABPA, TAF1, MAX, CREB1, UBTF, ATF2
**ERG**	ETS	GGA(A/T)	N/A	AML	Abnormal embryonic erythropoiesis Thrombocytopenia Decreased leukocyte HSC cell number Pancytopenia	No differentiation - specific role	TMPRSS2, AR, SPI1, RUNX2, CBFB	SPI1, TAF1, YY1, MAX, NFYB, UBTF, BRCA1, E2F1, MYC

Candidate inclusions to BMF/HM predisposition gene lists, ranked strong due to known role in hematopoiesis, previous disease association, pathogenicity predictor scores, and gene ontology. TF demonstrates similarities to known BMF/HM predisposition genes in reported human/mouse phenotypes, roles in downstream hematopoietic differentiation (15, 18–21) and known interacting TF partners and target genes. Known interacting partners collated through STRING (12) and target genes through ChEA from Chip-X (13).

NA, not applicable.

ETV6 germline aberration due to rearrangements, fusions, mutations, or deletions resulting in monoallelic expression of ETV6, contributes to several types of myeloid and lymphoid malignant susceptibility, with approximately two-thirds being B-ALL (85, 91). ETV6 is involved in over 30 translocation partnerships in leukemia and MDS including fusions with PDGFRB, AML1, MN1, JAK2, ASC2, ABL2, BTL, ARNT, MDS2, PER1 and an ETV6-RUNX1 fusion seen in over 22% of childhood B-ALL (79, 85). Subsequent to the translocation in B-ALL cases, the wildtype ETV6 allele is often mutated or deleted implicating its tumor-suppressive function (79, 92). Ironically, other ETS TFs involved in hematopoiesis (e.g., ERG, FEV) are also seen in non-hematological malignant fusions (ERG/TMPRSS2, ERG-EWS, FEV-EWS) (i.e., prostate cancer) as well as hematological malignant fusions (e.g., TLS/FUS-ERG and FLI1-EWS seen in myeloid leukemia and Ewing’s Sarcoma, respectively) (93). Germline ETV6 pathogenic variants phenocopy RUNX1 germline pathogenic variants in terms of platelet defects, heightened HM predisposition and their association with a poorer overall survival but, unlike RUNX1, clonal hematopoiesis has not been reported in ETV6 carriers (94, 95). Experimental studies including RNA sequencing indicate that despite patients harboring germline ETV6 or RUNX1 pathogenic variants having similar clinical phenotypes, distinctive molecular mechanisms occur to generate haploinsufficiency (94).

The majority of clinically reported ETV6 and FLI1 loss-of-function (LOF) pathogenic variants (ClinVar) cluster in the highly conserved ETS DNA binding domain (85). In the normal population, both ETV6 and FLI1 loss-of-function variants are not well tolerated (gnomAD, LOEUF 0.12 and 0.09, respectively) (29), consistent with them being pathogenic. (**Table 2**, **Figure 3**) Notably, because of the high conservation of their ETS domains, in regions of similarity, genetic changes may impact on structure and hence function such that variants demonstrated to be pathogenic in one ETS TF are likely also to be pathogenic at the corresponding amino acid position in another. Interestingly, pathogenic variants at 4 amino acids in FLI1 (ClinVar; p.R324W, p.R337Q/W, p.R340H/P, p.Y343C) have identical amino acids in wildtype ERG, ETV2, FEV and ETS1; identification of similar changes at the corresponding amino acids in these or other ETS TFs would be predicted to have similar deleterious effects.

Functional evaluation of germline ETV6 and FLI1 variants from leukemia and thrombocytopenia patients suggests that the substitution of conserved residues disrupts the protein’s general function. Using *in vitro* biochemical assays, it was found that damaging ETV6 variants resided within, or caused truncation of, the ETS domain, exhibiting significant impairment of transcription repression, a decreased ability to bind DNA and a loss of nuclear localization (76, 79, 85). Consistent throughout literature, pathogenic FLI1 variants also repress transcriptional activity in validated FLI1 target genes including GP9, exhibiting predominantly cytoplasmic localization and in contrast, result in large, fused platelets that have electron-dense α-granules, characteristic of the Paris-Trousseau syndrome (78, 80, 81).

The oncogenic potential of ETS TFs PU.1, ERG and ETS2 have been well characterized, making them convincing candidate genes for BMF and/or HM predisposition (**Table 4**, **Figure 3**); however, none are yet an established disease gene nor hold strong or definitive (ClinGen) gene-disease relationships. The importance of these TFs in normal hematopoiesis has been well established, both in lymphoid and myeloid lineages, however, studies have suggested these same TFs may also have a potential role in leukemogenesis. Cohort studies have identified the overexpression of ERG and ETS2 as a biomarker correlated with an adverse clinical outcome in AML patients, with ETS2 hypothesized to induce apoptosis (in the presence of p53) and ERG crucial for leukemic maintenance (96–100). Conversely, creating a model of hypomorphic PU.1 established a correlation between low PU.1 expression and AML (101, 102). This suggests that increases or decreases in the critical threshold levels of various ETS TFs may contribute to AML pathogenesis.

Like many other HM TF predisposition genes, the mechanisms of germline ETV6 and FLI1-mediated leukemogenesis remains poorly understood. Although, their direct associations with other important hematopoietic TFs such as with GATA2 and RUNX1 (**Table 1**) may highlight a mechanism by which missense variants disrupt the expression pattern of TFs within the normal hematopoietic network (12, 13, 96)

### The IKAROS family of BMF/HM predisposition TFs

The IKAROS protein family (IKZF) are master mediators of cell differentiation and function *via* DNA binding (ZF1-4) and dimerization domains (ZF5-6) which orchestrate transcriptional repression and/or activation of a large number of genes. IKZF1 and IKZF3 encode for IKAROS and AIOLOS proteins, and amongst other hematopoietic roles, are critical regulators of lymphoid development and differentiation. Germline variants in IKZF1 and IKZF3 result in a broad range of phenotypes including hematological ones which include immunodeficiency disorders (103) and most commonly lymphoid leukemias (104, 105). While dysfunctional IKZF2 is implicated in B-ALL and T-cell lymphoma, only IKZF1, IKZF3 and IKZF5 are formally recognized as BMF and/or HM predisposition genes (104, 106, 107). Interestingly, all three Ikaros BMF/HM predisposition genes are not highly expressed in hematopoietic cells in normal hematopoiesis or AML, indicating no correlation between expression patterns and predisposition to BMF or malignancy. (**Figure 2**) The potential of causal germline genetic variants to be discovered in other Ikaros family members (*i.e.*, IKZF2 and IKZF4) remains plausible, with high constraint scores (average pLI; 0.93, **Table 2**, **Figure 3**), despite IKZF4 poorly or not expressed during hematopoiesis (56).

Observed reoccurrence, familial segregation, and functional evaluation of IKZF1 variants enabled its identification as a HM predisposition gene. Somatic IKZF1 genetic aberrations have adverse effects on clinical outcomes (overall survival and relapse-free survival) and several molecular mechanisms have been implicated as the mode of IKAROS deficiency in both autoimmune deficiency syndromes and HM manifestation (108, 109). These include missense variants at DNA contact residues that are still able to dimerize with wildtype IKAROS but are unable to bind target DNA (103, 110), haploinsufficiency with deletion of one IKZF1 allele (103), differential expression in gene networks involved in cancer, cell signaling, apoptosis, and hematopoiesis (111) and transcription, dimerization, subcellular localization, and cell adhesion LOF (107, 108). Interestingly, somatic mutations in IKZF1 are recurrently detected in IKZF1 germline carriers with B-cell precursor ALL, suggesting that cells carrying the germline variant favor acquisition of a second (i.e., biallelic) IZKF1 transforming event (112). Curiously, the core consensus DNA binding motif for IKZF TFs is “GGAA” which is similar to that of ETS TFs, although the surrounding nucleotides confer greater specificity for specific IKZF and ETS TFs. Hence, at some binding sites there may be competition or synergism between some or many of these TFs depending on their expression levels and DNA promoter/enhancer contexts. Interestingly, in T-ALL cells, IKZF1 binding sites across the genome are most closely associated with FLI1 (similar GGAA consensus) and RUNX1 binding sites (113). Consistent with this, germline RUNX1 variants can also predispose to T-ALL as well as other lymphoid malignancies.

IKZF proteins form homodimers and heterodimers *via* their two C-terminal ZFs to initiate nucleosome remodeling and enable their pleiotropic roles in various hematopoietic cells. It is this phenomenon that sparks the possibility that aberration in any IKZF gene may contribute to leukemogenesis by perturbing the transcriptional networks of IKZF genes normal functions including lymphoid maturation, tumor suppression, cell-cycle regulation, kinase signaling and chromatin modification (108). Early reports associated germline IKZF1 deletions with poorer treatment responses and unfavorable outcomes (114–116), while more recent studies found the converse to be true, with beneficial responses to induction therapy (117).

### PAX5, MECOM, GATA1 and STAT3 BMF/HM predisposition TF genes

PAX5, MECOM and GATA1 are also critical contributors to cooperative transcriptional networks involved in hematopoiesis and included on germline targeted sequencing panels for BMF and/or HM susceptibility. These TFs demonstrate extremely low tolerance to LOF, with pLI scores all above 0.95, domain constraints scored intolerant or higher (30), and limited LOF seen in databases reflecting the normal population (gnomAD) (**Table 2**, **Figure 3**) (29). The oncogenic potential of these TFs is established by impactful somatic lesions, malignant fusions, and embryonic lethality.

PAX5 is a master regulator of lymphopoiesis, being required for normal B cell development and differentiation including B-lineage commitment, maintenance of B cell identity and VHDJH recombination. Somatic PAX5 aberrations are evident in ~30% of sporadic B-ALL cases (118) with mechanisms including copy number alterations (often deletion including -9/-9p), translocations generating fusion proteins that retain the DNA binding paired domain and nuclear localization signal of PAX5 (results in a fusion TF that acts as a dominant negative), intragenic amplification (including direct head-to-tail concatenation of exons 2 to 5 resulting in an extra 4-5 copies of the DNA binding and octapeptide domains), disruption to normal alternative splicing and isoform levels, and point mutations (119). The majority of these are believed to decrease PAX5 expression and/or transcriptional activity causing both repression and activation of downstream target genes (120). In almost all B-ALL cases with point mutations, there is a concomitant loss of the wildtype allele. However, complete loss of PAX5 activity is not seen in B-ALL suggesting that a residual amount of activity is required for the disease phenotype.

PAX5 has few known germline aberrations. The p.G183S missense variant, reported in three unrelated families and 2 sporadic cases, is located in the octapeptide domain, and strongly linked to pre-B cell leukemogenesis with impaired capacity for transcriptional activation and/or repression (118, 121–124). PAX5 variants follow autosomal dominant transmission with variable penetrance, suggesting it is likely that additional secondary or tertiary predisposing elements occur in these families, particularly somatic loss of the wildtype (WT) PAX5 allele due to aberrations of chromosome 9p (118, 121). Incomplete penetrance seen in PAX5 mutated families is likely explained by the synergistic effects of these secondary or tertiary events, including environmental factors since, in heterozygous Pax5 mice, infection exposure mediated HM development (123). Interestingly, only one other PAX5 germline missense variant (p.R38H) has been linked with leukemic predisposition (B-ALL). Curiously, 2 of 3 germline (p.R38H) cases acquired a PAX5 (p.R140L) mutation (125) which is in keeping with somatic p.R38H and p.R140L mutations co-occurring in a biallelic fashion in 10/11 B-ALL patients harboring a p.R140L mutation (125). Functionally, p.R38H is not able to regulate PAX5 target genes nor trigger B-cell differentiation, maintaining PAX5 cell growth properties only partially, without overt dominant-negative effect on the normal PAX5 function (125, 126). Presumably, the combination of these 2 concurrent variants provides PAX5 activity at levels highly conducive to HM. Somewhat like germline RUNX1 and CEBPA-driven HM, and even more so, in germline PAX5-driven B-ALL, the WT allele of PAX5 is almost always deleted or mutated in some way to abrogate PAX5 activity from this allele, in addition to the perturbation due to the inherited germline variant allele (119).

MECOM is a ZF TF gene which through differential splicing encodes three protein isoforms: MDS1, EVI1 and fusion protein MDS1-EVI1 (127). Typically, EVI1 has repressor functions while MDS1-EVI1 acts as a transcriptional activator. MECOM is a stemness gene, required for long-term HSC survival and is essential for regulating both embryonic and adult HSCs, by directly regulating GATA2. Germline MECOM variants cause heterogenous BMF syndromes and only recently was identified as a predisposition gene to inherited HM (128). Haploinsufficiency of MECOM, leads to loss of HSC within the first few months of life, *via* defects in stem cell self-renewal proliferation and repopulation capacity, causing severe neonatal BMF (129, 130). The location of pathogenic germline variants is somewhat associated with the phenotypic variability observed in patients. Germline variants causing haploinsufficiency including nonsense, frameshift variants and *de novo* microdeletion (covering entire locus or just the MDS1 coding regions) causes congenital thrombocytopenia but not skeletal abnormalities (131) whereas missense variants in the ZF2 domain affecting the MDS1-EVI1 and EVI1 transcripts cause limb defects and thrombocytopenia including radioulnar synostosis with amegakaryocytic thrombocytopenia 2 (RUSAT2; OMIM #616738). These missense variants cause partial LOF or gain-of-function (GOF) (132). Missense mutations in MECOM typically cluster within the C-terminal ZF domain which contains an ETS-like motif, affecting the protein folding stability or DNA-binding amino acid residues. Transactivation studies have demonstrated varying effects of these missense variants on transcriptional regulation with a possible dominant-negative effect on TF AP-1 (Jun/Fos) signaling, and partial LOF of TGF-beta signaling (132). The C-terminal ZF domain also includes an oligomerization domain required for homodimerization with EVI1 and interaction with RUNX1.

Chromosomal rearrangements targeting MECOM are relatively common in sporadic myeloid leukemias. These include inversion inv(3)(q21.3q26.2) and translocation t(3;3)(q21.3;q26.2) which result in EVI1 overexpression (133). Translocation t[3;12] leading to RUNX1-EVI1 fusion and t[3;12] resulting in ETV6-EVI1 fusion both result in EVI1 overexpression. Interestingly, and not surprisingly, both the inv(3)(q21.3q26.2) and t(3;3)(q21.3;q26.2) rearrangements converge on the GATA2 gene, resulting in bringing the GATA2 enhancer close to the MECOM gene which leads to ectopic MECOM expression and GATA2 haploinsufficiency (134). The RUNX1-EVI1 fusion preserves the DNA-binding RUNT domain of RUNX1, with loss of the transactivation domain. Expression of RUNX1-EVI1 in progenitor cells committed to the hematopoietic lineage results in activation of a pan-lineage hematopoietic gene expression program, and a loss of regulation of vascular differentiation programs and partial cell-cycle arrest, likely affecting the terminal differentiation potential of multipotent progenitors (135). This fusion protein acts as a dominant-negative, suppressing the transactivation capacity of WT RUNX1 (136).

GATA1 is a ZF TF and a master regulator of erythroid development. GATA1 variants cause a range of hematological phenotypes, including X-linked thrombocytopenia with or without dyserythropoietic anemia, with several different syndromic manifestations including Diamond Blackfan Anemia, β-thalassemia and congenital erythropoietic porphyria. As is observed with other TFs involved in hematological disease, location within the gene and variant type is associated with clinical presentation. As GATA1 is located on the X-chromosome, loss-of-function variants are typically more severe in males with females often being asymptomatic although presentation is impacted by levels of X-chromosome inactivation skewing. (**Table 1**) (137–139) Two main classes of GATA1 variants exist: 1) splicing or start-loss, which lead to expression of a shorter isoform protein (i.e. GATA1s - without N-terminal transactivation domain), which can give rise to moderate to severe anemia, neutropenia and/or DBA-like phenotypes (138, 140, 141) and 2) missense variants in exons 3 and 4, most commonly in the N-terminal ZF domain which mediates the interaction with co-factor Friend of GATA1 (FOG-1) giving rise to cytopenia-related phenotypes (137, 138, 142). These missense mutations have been shown to affect the interaction with FOG-1 or the DNA binding ability of GATA1 (139, 143). GATA1 polymorphisms have also been shown to act as genetic modifiers in disorders caused by variants in GATA1-dependent genes (144). Acquired GATA1 mutations resulting in expression of GATA1s are common in Down Syndrome patients with trisomy 21, causing transient abnormal myelopoiesis that may spontaneously resolve or progress to AML. Interestingly, two families with germline GATA1 variants resulting in expression of GATA1s had several family members who developed acute megakaryoblastic leukemia with acquired trisomy 2, suggesting the order of mutation acquisition is not important (145).

STAT3 is a TF critical for appropriate cell proliferation, inflammation, differentiation, survival, and an important mediator of the innate and adaptive immune response (146, 147). STAT3 is the most common member of the STAT protein family to be mutated in hematopoietic cancers. GOF and LOF germline variants have been identified and are observed to give rise to immunodeficiency, autoimmune and cancer phenotypes. Germline STAT3 LOF variants are responsible for autosomal-dominant hyper–immunoglobulin E syndrome while GOF/activating variants are causal for early-onset multiorgan autoimmunity (148) including lymphoproliferation and pediatric large granular lymphocytic leukemia (LGL) including neutropenia, thrombocytopenia (149). Germline variants in GOF STAT3 have been identified in multiple domains including the all-alpha, DNA binding, SH2, and C-terminal transactivation domain of the protein (149). The high rate of concurrent self-reactive autoimmunity and LGL in carriers of STAT3 GOF variants has led to the concept that they develop *via* similar molecular mechanisms (150–152). Other common syndromic features include interstitial lung disease, diabetes, and postnatal growth failure (153). Somatic GOF variants in STAT3, which cluster in the Src homology 2 (SH2) domain, are associated with granular lymphocytic leukemia, myelodysplastic syndrome, and aplastic anemia (150, 154). STAT3 is activated by multiple signaling pathways, beyond the canonical JAK‐STAT pathway, including *via* cytokine signaling, receptor tyrosine kinase signaling, and G-protein coupled receptor signaling, to activate or repress transcription of target genes, explaining the broad functional role of STAT3 in regulating cellular functions (155, 156). Given the GOF are observed in all functional domains of the protein, it is thought that different variants in different locations of the protein will exert GOF effects *via* different steps of the signaling pathway for example, dimerization, DNA-binding, nuclear shuttling, or phosphorylation which could account for the phenotypic variability (155).

## Biological mechanisms of BMF and/or HM predisposition TFs

TFs are key regulators of hematopoietic reprogramming, with each TF exhibiting specific temporal and spatial expression patterns during lineage differentiation. The TF sub-networks are tightly regulated, functioning in specific cell types to modulate hematopoiesis. As a result of this, and potentially with the influence of both internal and external stressors, the biological mechanisms by which distinct TF aberrations functionally influences normal hematopoiesis is highly variable. Biological models are often relied upon to elucidate the role of TFs in hematopoietic pathways, including identifying interacting partners and target genes, to gain insight into how aberrations contribute to the development of disease. It is possible that a variant may only display activity under certain physiological or pathological contexts and investigating its function in traditional biological models (e.g., iPSC cells, primary cell cultures, animal models) may not unveil salient information without knowledge of these external factors. Germline TF pathogenic variants add another layer of complexity by their non-autonomous dysregulation in hematopoietic cells, and therefore, functional consequences may only become apparent in heterocellular populations (157). Nevertheless, biological models can be very useful in many contexts. Given other comprehensive reviews on penetrant HM predisposition genes RUNX1, CEBPA, PAX5 (9, 10, 158) and the fact that the ZF and the ETS DNA binding domains are two of the most prominent functional domains associated with BMF and/or HM predisposition TFs, this section will discuss the biological mechanisms of TFs containing these functional domains.

### Zinc finger BMF/HM predisposition TFs

The GATA and IKAROS families and MECOM isoforms all share structural ZF domains which function in both DNA binding, domain structure and protein: protein interactions. However, despite relatively high amino acid homology between individual members of each group, they display complex spatial, temporal and cell-type differences in expression, binding partners, role in regulation of hematopoietic networks, and underlying biological mechanisms contributing to BMF and/or HM. Gata1(-/-), Gata2(-/-), Evi1(-/-), and Ikzf1(-/-) null mice all display embryonic lethality due to hematopoietic defects, highlighting their importance in hematopoiesis. Further, in heterozygosity, milder hematopoietic phenotypes demonstrate haploinsufficiency on prenatal definitive hematopoiesis and the function of adult HSCs (159–165).

GATA2 is integrated into a regulatory network that includes DNA-binding, interaction with numerous cofactors, genetic and epigenetic transcriptional regulation of hundreds of GATA2 target genes, and response to cellular and extracellular signals controlling its expression in positive and negative feedback loops. GATA2 directly regulates the expression of multiple target genes such as RUNX1, TAL1, SPI1, FLI1 and LMO2 (166), and different enhancers regulate specific spatial and temporal hematopoiesis. This regulation varies greatly in different biological systems and may only operate in restricted physiological and/or pathological contexts and states (e.g., progenitor cell vs mature cell or steady state vs stressed state) (167, 168). Consequently, germline variants in GATA2 may exhibit hypomorphic characteristics under some conditions, but not under others. For instance, patients with GATA2 deficiency syndrome may develop different syndromic features under different stresses such as BMF or lymphedema with persistent infections or inflammation, myeloid malignancy with biological (e.g., cytosine deamination with aging) or chemical-induced acquisition of pathogenic mutations, hearing loss due to disruption in development of ear structures due to aminoglycoside antibiotic or stochastic events leading to urinary system malformation (169). A delicate balance of ebbs and flows of GATA2 activity is required to establish and maintain GATA2-dependent regulatory networks throughout normal and stress-induced hematopoiesis or other bodily systems (168).

The inhibition of GATA2 has been shown to be linked to biological traits of leukemogenesis in multiple systems such as human cord blood where knockdown of GATA2 markedly reduces colony forming cell growth capacity and lineage-specific colony forming cells (170). Gata2 depletion also reduces multi-lineage potential in mice, as well as HSPC cell numbers (171). Haploinsufficiency does not completely diminish hematopoietic programming but reduces the capacity of HSPCs to complete HSC maturation during the endothelial to hematopoietic transition, where Gata2(+/-) is able to initiate the process, but unable to fully execute it (172). Knockout mice exhibit near entire loss of HSCs, primitive progenitor cells and committed myeloid and erythroid progenitors (170), whilst GATA2(+/-) mice show markedly reduced HSC numbers suggesting dose-dependence. However, due to spatial and temporal expression patterns, some cells still achieve their threshold of GATA2 activity, and thus their target genes may still be appropriately regulated. Similarly, in GATA2-deficient iPSC systems, hematopoietic progenitors (HPCs), and erythroid and granulocyte progenitors are markedly reduced, despite having little effect on specification of mesodermal and endothelial lineages at pre-hematopoietic fates (173). However, like haploinsufficient mice, some patient-derived iPSCs with GATA2 variants (e.g., p.R361H) were able to retain sufficient residual GATA2 activity for normal hematopoietic development (173) as is seen in asymptomatic carriers.

GATA1 plays an indispensable role for the downstream differentiation of various hematopoietic lineages, including myeloid stem cells, megakaryocytes, erythrocytes, mast cells, platelets, and dendritic cells. (**Figure 2**, **Table 1**) GATA1s (GATA1 “short” - lacks N-terminal domain) has been extensively used as a biological tool to investigate the effect of GATA1 aberration on hematopoiesis, where LOF GATA1 initiates the arrest of both primitive and definitive erythropoiesis by inducing apoptosis in erythroid progenitors and phenotypically displays BMF syndromes (e.g., disordered terminal platelet maturation) without manifesting into leukemia (162, 174). Interestingly, mimicking the acquisition of *de novo* GATA1 mutations in primary human HSPCs, the same loss of GATA1 has the opposite effect on megakaryocytes, causing fetal (not neonatal or adult) megakaryocytic progenitor hyperproliferation, where megakaryocytes fail to undergo differentiation but expand dramatically, by potentially hijacking the myeloid mechanism to promote this proliferation (174–176). This abnormality has also been linked with a myelofibrotic phenotype in mice, and the (GATA1low) mice has been extensively used a biological tool for a targeted therapeutic pathway, using and Ruxolitinib and a monoclonal antibody (RB40.34) to improve effective hematopoiesis in BM by restoring spleen architecture and reducing fibrosis in the BM (177).

The role of GATA1 in leukemogenesis (differentiation block, proliferation) has been shown primarily in trisomy 21 models where spatiotemporal intensity of GATA1s expression contributes to pathological phenotypes. For example, point mutations in trisomy 21 iPSCs show GATA1s impairs megakaryocytic differentiation, by inducing the emergency of megakaryoblasts with major platelet and a-granule formation impairments (178). Conversely, acquired trisomy 21 has been reported in a patient carrying a germline GATA1 variant causing increased GATA1s, and associating with chronic anemia and thrombocytopenia as well as transient abnormal myelopoiesis, although the latter is likely a result of the trisomy 21 (179).

Mediated through the two ZF domains, the duality of GATA1 as both an activator and repressor results in the formation of several distinct complexes involved in hematopoiesis (180), with a variety of partners including LMO2 (181), RUNX1 (182), FOG1 (183), TAL1, GFI1B, ZFP143, ETO2 (184), LDB1 (182), FLI1, EKLF, HDAC5 and PU.1 (185). Study of these interactions may provide insight into GATA1-mediated predisposition. For example, Runx1 expression in zebrafish cooperatively promotes primitive hematopoiesis with GATA1 (186), and methylation of the Gata1 locus (by recruitment of DNMT1 and the GATA1 methylation determining region) prevents GATA2-mediated GATA1 activation in HSPCs (187).

Recent studies have suggested that environmental factors may play a role in the development of GATA1 mutations, as epigenome-wide association studies have linked a metastable epiallele (VTRNA2-1) to a differentially methylated GATA1 region associated with Down Syndrome (188). Additionally, the ZF domain of GATA1 is structurally favorable for interactions with other proteins, such as ASIII, which has been shown to suppress GATA1 (and GATA2) function (189). This suppression may mediate differentiation blocks that are associated with leukemia.

MECOM mirrors the regulatory effect of GATA2 on normal hematopoiesis, demonstrating a vital role in the differentiation of HSCs directly through upstream regulation of GATA2. Inherently, Evi1(-/-) mice also show marked reduction of HSCs through simultaneous reduction of GATA2 expression seen in these models (190), and predictably, reconstituting EVI1 expression upregulates GATA2 expression and rescues HSC differentiation ability (191, 192). Heterozygous CRISPR edits of MECOM engrafted bone marrow into mice also reduced HSC production ~2 fold, but found no detectable differences in lymphoid, erythroid, megakaryocytic or monocytic lineages (192). The ZF domains enable association in DNA repair, chromatin remodeling and transcription, including with downstream TF targets (i.e., SPI1, GATA1/2, RUNX1 and CEBPA) driving the MECOM regulatory network (193, 194). Interestingly, amongst RUNX1, GATA2 and JUN, CHIP-seq data identified the most enriched motif in HSPCs is the ETS motif, which can be bound by ERG, ETV6, ETV2 and FLI1, despite highly enriched TF occupancy by FLI1, RUNX1 and GATA2 (192). Single-cell transcriptomics exemplifies the consequence of MECOM disruption (CRISPR-mediated) by dysregulating over 700 genes including key factors expressed during hematopoiesis (192).

The function of EVI1 is critically modulated by posttranslational modifications including phosphorylation, sumoylation, ubiquitylation, and monomethylation (195). Constitutive expression of EVI1 through bone marrow transplantation in mice recapitulates phenotypes resembling MDS. (**Table 5**) Although none manifest into leukemia, EVI1 induces delays in HSC and erythroid differentiation which in later stages (10-12 months) lead to hematopoietic failure and death (196). The upregulation of MECOM and consequent down regulation of network targets is associated with poor prognosis in AML (192). Hence, EVI1 dysregulation may be a contributor in AML risk stratification (197) and may be a target for therapeutic intervention. For example, knockdown of EVI1 in K562 cells shows a greater sensitivity to therapeutic drugs (e.g., Imatinib) and amongst other targets (PTGS1, COX-1/2), prevents expression of a gene that affects platelet regulation (ITGA2B) (198).

**Table 5:** Hematological-related mouse phenotypes found in BMF/HM predisposition genes including strong candidate genes and other candidate genes.

	TF gene symbol	MGI Mouse Phenotype (Hematological)
		Phenotype
**Known BMF/HM TFs**	**Runx1**	**- abnormal** hematopoiesis-related cell morphology/development/number, hematopoietic differentiation, and spleen/thymus size/morphology - Thrombocytopenia
	**Cebpa**	- enlarged spleen **- abnormal** definitive hematopoiesis, myelopoiesis, granulocyte, leukocyte common myeloid progenitor and bone marrow cell morphology and myeloid leukocyte - increased erythroid progenitor, hematopoietic, neutrophil, granulocyte, monocyte cell number and decreased hematocrit
	**Gata2**	**- abnormal** embryonic/definitive hematopoiesis, blood cell/megakaryocyte morphology/development and stress erythropoiesis/hematopoiesis - decreased common myeloid/erythroid progenitor cell number - anemia
	**Pax5**	**- abnormal** lymphopoiesis, class switch recombination, B/T cell number/differentiation/physiology, immunoglobulin heavy chain V(D)J recombination and macrophage, monocyte and leukocyte cell number - decreased immunoglobulin and IgA, IgD, IgG1, IgG2a, IgG2b, IgG3, IgG, IgM levels
	**Mecom/Evi1**	**- abnormal** neutrophil differentiation, common myeloid progenitor cell morphology/number, bone marrow cell morphology/development, platelet, HSC and fetal liver HPC morphology and leukocyte, HSC, precursor and B-cell numbers - thrombocytopenia
	**Stat3**	**- abnormal** thymocyte, T cell apoptosis/proliferation, thymocyte, neutrophil, dendritic cell, T- granulocyte, macrophage, monocyte and leukocyte cell number, T cell, macrophage differentiation/physiology and IgE, IgG1 and IgG3 levels
	**Gata1**	**- abnormal** embryonic erythropoiesis, definitive hematopoiesis and megakaryocyte differentiation, spleen, common myeloid progenitor cell, megakaryocyte, proerythroblast, erythrocyte, platelet, mononuclear, spleen, bone marrow cell and erythroid progenitor morphology, erythropoiesis, myelopoiesis, thrombopoiesis and extramedullary hematopoiesis, bone marrow, megakaryocyte, erythroid progenitor, eosinophil, erythrocyte, neutrophil, leukocyte, lymphocyte, osteoclast and splenocyte cell numbers, and hemocrit - anemia, anisocytosis, poikilocytosis, polychromatophilia and thrombocytopenia
	**Etv6** **(Tel)**	**- abnormal** megakaryocyte, B cell differentiation, definitive hematopoiesis, erythropoiesis and lymphopoiesis, spleen, common myeloid progenitor, lymphocyte progenitor cell and myeloblast morphology, erythroid progenitor, erythrocyte, neutrophil cell, B cell, lymphocyte and HSC cell numbers and bone marrow cell physiology - myeloid hyperplasia and thrombocytopenia
	**Fli1**	**- abnormal** megakaryocyte, B cell differentiation, erythropoiesis, thrombopoiesis and hematopoiesis, thymocyte, B cell, megakaryocyte, erythroid progenitor, erythrocyte, granulocyte, NK and monocyte cell numbers, spleen and megakaryocyte, proerythroblast and leukocyte morphology - decreased B cell proliferation, hematocrit and IgG1 and IgG3 levels - anemia and thrombocytopenia
	**Ikzf1**	**- abnormal** B cell apoptosis, granulocyte, macrophage and B/T cell differentiation, B/T cell proliferation, thymus and spleen, definitive hematopoiesis, erythropoiesis, lymphopoiesis and myelopoiesis, common myeloid progenitor cell morphology, B/T, bone marrow, erythroid progenitor, basophil, erythrocyte, granulocyte, dendritic, NK cell, macrophage and splenocyte cell numbers, erythroid progenitor cell, eosinophil, erythroblast and NK morphology, and natural killer cell mediated cytotoxicity. - myeloid hyperplasia, extramedullary hematopoiesis and anemia - decreased hematocrit, hemoglobin content and IgG3 and IgG levels
	**Ikzf3**	**- abnormal** B/T cell proliferation and differentiation, spleen, neutrophil, B- cell, lymphocyte and leukocyte cell numbers and IgE, IgG1, IgG2a, IgG2b, IgG3 and IgM levels - thrombocytopenia
	**Ikzf5**	**- Abnormal** IgA and IgE levels - Epistaxisl, Joint hemorrhages, Persistent bleeding after trauma, Bruising susceptibility, Petechiae, Anemia of inadequate production, Poikilocytosis, Acanthocytosis, Leukocytosis and Thrombocytopenia - Increased megakaryocyte colony forming unit counts
**Strong candidate BMF/HM TFs**	**Gata3**	- increased T cell apoptosis and decreased IgE levels **- abnormal** thymus, thymocyte, T- and eosinophil cell numbers, definitive hematopoiesis, and T cell differentiation/morphology
	**Spi1** **(Pu.1)**	**- abnormal** granulocyte, neutrophil, macrophage, and B/T cell differentiation, thymus and spleen, blood cell, bone marrow common myeloid progenitor, T-cell, proerythroblast, granulocyte, microglial, monocyte and spleen morphology, definitive hematopoiesis, erythropoiesis, leukopoiesis and myelopoiesis, B/T-, bone marrow, erythrocyte, granulocyte, neutrophil, Lymphocyte, macrophage, HSC cell numbers and HSC physiology. - increased hematopoietic stem cell proliferation, mean corpuscular volume and IgM level, and decreased thymocyte number and hematocrit - absent common myeloid progenitor cells and anemia and extramedullary hematopoiesis
	**Erg**	**- abnormal** embryonic erythropoiesis, spleen morphology and leukocyte, HSC and B cell numbers (decreased) - thrombocytopenia and pancytopenia
**Other candidate BMF/HM TFs**	**Etv2**	**- abnormal** spleen, embryonic erythropoiesis, definitive hematopoiesis, myelopoiesis, bone marrow and myeloblast morphology/development, common myeloid progenitor, bone marrow, erythroid progenitor, leukocyte and HSC cell number and absent erythrocytes - anemia
	**Elf1**	**- abnormal** B cell differentiation and B cell physiology
	**Fev**	No Hematological phenotype reported
	**Ets1**	**- abnormal** B cell, thymocyte, splenocyte apoptosis, B/T cell proliferation, thymus and spleen, lymphopoiesis, B cell differentiation, B/T-, plasma, NK, leukocyte cell numbers, leukocyte, NK cell physiology and T cell activation - impaired natural killer cell mediated cytotoxicity
	**Ets2**	No Hematological phenotype reported
	**Ikzf2**	**- abnormal** regulatory T cell physiology
	**Ikzf4**	- decreased T cell proliferation and increased immunoglobulin level **- abnormal** spleen and B/T cell number
	**Gfi1b**	**- abnormal** megakaryocyte differentiation and erythropoiesis, blood, bone marrow cell morphology/development, erythroid progenitor, erythrocyte, neutrophil, lymphocyte, HSC, splenocyte cell numbers, megakaryocyte progenitor and embryonic erythrocyte cell morphology and hemoglobin content (decreased) - anemia and thrombocytopenia
	**Tal1** **(SCL)**	**- abnormal** embryonic hematopoiesis and erythropoiesis, thymus and spleen, common myeloid progenitor, bone marrow, leukocyte, and HSC cell numbers and bone marrow, megakaryocyte, erythroid progenitor and proerythroblast cell morphology - anemia, anisocytosis and thrombocytopenia - decreased hematocrit and hemoglobin content, mean corpuscular hemoglobin concentration and mean corpuscular volume
	**Lyl1**	**- abnormal** B/T cell number/differentiation and hematopoietic stem cell physiology - thrombocytopenia
	**Lmo2**	**- abnormal** embryonic hematopoiesis, thymus and spleen, common myeloid progenitor and megakaryocyte progenitor cell morphology, erythrocyte, granulocyte and monocyte cell number - decreased hemoglobin content, increased mean corpuscular volume - thrombocytopenia
	**Meis1**	- decreased common myeloid progenitor, megakaryocyte, platelet cell number **- abnormal** bone marrow and megakaryocyte progenitor cell morphology - increased hematopoietic stem cell number
	**Tcf12**	- thymus hypoplasia **- abnormal** thymocyte B/T cell number and morphology - arrested T cell differentiation
	**Gfi1**	**- abnormal** granulocyte, monocyte, NK and T-cell differentiation, T cell proliferation, thymus, thymocyte, T/B-cell neutrophil, granulocyte, monocyte and HSC number, definitive hematopoiesis, lymphopoiesis and myelopoiesis, neutrophil and monocyte morphology and NK cell and T-helper physiology
	**Fos**	- increased macrophage apoptosis **- abnormal** osteoclast differentiation, thymus and spleen, thymocyte, T/B-cell, granulocyte, splenocyte and osteoclast cell number, lymphopoiesis and macrophage physiology
	**Hoxa9**	**- abnormal** common myeloid progenitor cell morphology, bone marrow and HSC cell number and spleen
	**Fosb**	No Hematological phenotype reported
	**Foxc2**	No Hematological phenotype reported
	**Eklf (Klf1)**	**- abnormal** spleen, erythropoiesis, hematopoiesis and erythroblastosis, erythrocyte morphology and cell number - decreased hematocrit, hemoglobin content, mean corpuscular hemoglobin - anemia, anisocytosis, poikilocytosis and polychromatophilia
	**Nfkb1**	**- abnormal** spleen, B/T cell proliferation, bone marrow cell morphology/development, erythrocyte, B/T-, NK, myeloid dendritic, osteoclast and leukocyte cell number, lymphocyte, B/T cell and macrophage physiology, IgA, IgE, IgG1, IgG3, IgG, IgM and immunoglobulin levels and CD4-positive, alpha-beta T cell physiology - myeloid hyperplasia and decreased mean corpuscular hemoglobin concentration

Mouse phenotypes collated through Mouse MGI (25–27).

NA, not applicable.

A commonality in their multiple ZFs as well as their belonging to the same TF family, may imply that the mechanisms underlying IKZF-1, -2, and -3 contributions to BMF and/or HM are comparable. However, IKZF TFs typically exhibit functional redundancy, meaning that the absence of one is often compensated by the presence of another (199). This can be well articulated by the phenotypic differences in IKZF1 mice. Homozygous Ikzf1 mice containing a missense mutation in ZF3 (p.H191R) result in embryonic lethality, whilst the same heterozygous mutation has normal numbers of B-cells, but reduced numbers of B-cell precursors in the bone marrow (103). Similarly, the lack of T and B cell progenitors as well as NK cells was a result of a deletion of the first 3 ZFs. Mice with a deletion of 3 N-terminal ZFs, however, have a high incidence of T-cell leukemia. Despite these mutations seemingly resulting in the dysregulation of IKZF1, mutations retaining the ability to form heterodimers with other IKZF family proteins reflect a less severe phenotype (199). Interestingly, a mouse model harboring an IKZF3 variant (p.G158R) was able to affect the binding of both IKZF1 and IKZF3 (199). Although homozygous IKZF3 and IKZF5 knockouts have been shown to be viable, (**Table 1**) complete loss of IKZF1 results in embryonic lethality, associated with defects in erythroid cells and an expansion of megakaryocyte progenitors, highlighting the importance of this TF in hematopoiesis over its familial counterparts (161).

### ETS BMF/HM predisposition TFs

Oncogenic transformation relies on dysregulated use of normal developmental pathways, and ETS TFs highly co-operative roles in normal hematopoiesis suggests that the impact of one ETS TF aberration may change the regulatory role of others. Interestingly, through the protein’s pointed domain, ETV6 has direct interaction with another known ETS TF BMF predisposition gene, FLI1 (200). This interaction has now been shown to alter tumor growth in Ewing’s Sarcoma patients, where the EWS-FLI1, an oncogenic fusion protein that drives tumor growth in 90% of patients, is constrained by a loss of ETV6 (201).

The central role of ETV6 as a ‘transcriptional suppressor’ makes its dysregulation unsurprising in HM. For example, co-binding of ETV6 and a protein complex mediating chromatid cohesion has been shown to lead to transcriptional repression of erythropoiesis-related genes, suggesting involvement in inhibition of erythroid differentiation in myeloid malignancies (202). Etv6(-/-) mice result in embryonic lethality with yolk sac angiogenic defects and perturbation of definitive hematopoiesis in bone marrow. (**Table 2**) (203) Interestingly, a mouse model of the most common ETV6 pathogenic variant outside of the ETS-domain, p.P214L (in mice Etv6P216L/wt), did not show homozygous lethality and the mice had no overt hematopoietic phenotype. In conditional knockout mice, thrombocytopenia is observed as the primary phenotype due to an increase in megakaryocytic colony forming cells implying a loss of ETV6 results in a terminal defect in megakaryocyte maturation (204). (**Table 5**) Modeling ETV6-associated leukemia commonly uses the ETV6-RUNX1 fusion protein. In mouse models, a low incidence of leukemia is seen, developing only after a long latency (205) and/or secondary genetic hits (206), and often results in inactive HSCs and loss of lymphoid progenitors (207).

Competitive transplant experiments revealed Etv6P216L/wt HSPCs had reduced lymphoid reconstitution potential. Specifically, Etv6P216L/wt mice showed impaired MPP4 hematopoietic progenitor populations with lymphoid potential (208). Transcriptome analysis of the MMP4 population from these mice suggested deregulation of inflammatory pathways as a consequence of the Etv6 variant, however no significant difference in cytokine or chemokines were identified in the BM. These mice also did not develop leukemia, suggesting additional models or stressors are required to achieve the full range of human disease phenotypes.

In patient-derived peripheral blood mononuclear cells, the lowering of ETV6 results in an increase in histone acetylation suggesting epigenetic changes are likely to contribute to hematopoietic dysregulation. Further, single cell RNA-seq of ETV6 variants p.P214L and p.R369Q revealed enrichment of interferon response genes across peripheral blood cell populations, and HDAC3 as an upstream master regulator (209) consistent with the ETV6/HDAC3 complex regulating the interferon response. Dysregulation of the complex in megakaryocytes impaired proplatelet formation. ETV6 variants (e.g., R369Q) in heterozygosity have also been shown to drive thrombocytopenia in iPSCs, with more, but less responsive megakaryocytes that are deficient in platelet formation, leading to fewer platelets (94).

FLI1 is the most highly expressed BMF and/or HM TF predisposition gene in both myeloid and lymphoid lineages during most stages of hematopoiesis (**Figure 2**), and directly regulates expression of various target genes within its broad target gene program, which may be the mechanism behind its BMF association. For example, FLI1s large transcriptional target program allows the protein to alter glycolysis, shifting the glycolytic balance to be more aerobic to enable robust cell division, and through the repression of the PKLR (pyruvate kinase) promoter, can initiate a block in erythroid differentiation (210). Despite its colocalization with GATA1/2 and RUNX1 (211), FLI1 has also been shown to cooperate with RUNX1 by co-binding to ETS-RUNX motifs, to restrain transcription factors that aid T-cell differentiation (212). Importantly, as a key regulator of T-cell differentiation programs, FLI1 deficiency does not diminish the T-cell population, but aids in safeguarding its transcriptional and epigenetic commitment (212).

Fli1 mouse models utilizing the deletion of its carboxy-terminal regulatory (CTA) domain showed impairment of megakaryocytic development and platelet number and function (213). Work showing a synergistic relation between FLI1 and GATA1 to regulate megakaryocytic genes (214), was later confirmed by the failure of aberrant FLI1 to recruit GATA1 to several megakaryocytic promoters (213). Similarly, in human-derived iPSCs, megakaryocyte and platelet defects were shown to be predominantly a result of FLI1 deficiency (215).

## Gene annotation and curation standards for TFs

As described above, core hematopoietic TFs including RUNX1, GATA2, GATA1 and MECOM play crucial roles in regulating multiple aspects of hematopoiesis and HSPC biology. Due to their central involvement, it is not surprising that genetic variation in these TFs can lead to differentiation blocks and perturbations in the regulatory networks that govern normal hematopoiesis, and thus lead to BMF and HM. While these genes are well known predisposition genes, it remains challenging to recognize other germline predisposition genes or variants in known genes especially if penetrance is low, phenotype expressivity is variable, frequency of pathogenic gene variants is very rare, or the variant exists in promoter/enhancer regions that are not characterized or linked to the causal gene.

Recurrence within families with a history of HM, BMF or a bleeding disorder, as well as identifying individuals that develop HM at a young age are strong predictors for moderate-highly penetrant germline predisposition, although there are more and more studies demonstrating that the lack of family history of a particular BMF or HM is not a great indicator that an individual is not a carrier of a germline predisposition variant (216, 217). Advancements in technology, accessibility and reduced costs associated with Next-Generation Sequencing (NGS) technologies has allowed for more routine investigation of a germline genetic cause in individuals and/or families. With increasing numbers of individuals being screened *via* NGS, undoubtedly this results in identification of novel variants and genes associated with these disorders. The American College of Medical Genetics and Genomics (ACMG) has been instrumental in developing guidelines for the interpretation of genetic variants, although these guidelines are developed for genes with a definitive role in the pathogenicity of the disease (218). Thus this can make it challenging to identify novel disease genes. Classification of variants according to ACMG criteria is based on the strength of available evidence including population frequency, computational data, functional studies, segregation data and relies on knowledge of disease mechanisms and clinical information (218). The criteria used to classify genetic variants means it is challenging to determine novel disease genes as it often relies on multiple probands with the same phenotype, associated segregation data and functional studies to validate the gene as disease-causing. In the context of predisposition to HM, additional contributing factors are not considered including ontology, environmental factors, and somatic data. Somatic mutations are a common molecular mechanism behind oncogenicity, and so the inclusion of this data to pathogenic classification should not be overlooked. Correct classification of genetic variants is crucial in disease management and treatment.

Estimating the number of genes missed by confining variant classification to a guidelines-based approach is complex, yet their existence can be speculated. From a discovery perspective such guidelines can be extrapolated to dealing not only with germline “variant of uncertain significance” (VUS), but also with “gene of uncertain significance” (GUS, for a gene not previously known to cause a certain phenotype) and “phenotype of uncertain significance” (PUS, for a phenotype not previously associated with a gene; that is, phenotype expansion for a gene). Ultimately, a collective weight of evidence is required to transition each of these out of the “uncertain” classification.

Incorporation of somatic data in variant curation can assist in determining the pathogenicity of a novel variant; for example, somatic DDX41 mutations are often detected in individuals with myeloid malignancy with a germline DDX41 variant (80% of cases) but vary rarely seen in the absence of a pathogenic germline DDX41 variant (219, 220). Progression of disease resulting from germline predisposition of penetrant genes (GATA2, RUNX1 and CEBPA) is often accelerated by the acquisition of unique sets of somatic gene aberrations affecting disease progression, clonal architecture, and treatment response (221). Somatic RUNX1 alterations (translocations and mutations) are frequently associated with MDS and AML and are considered responsible for leukemic progression in transformation from BMF into leukemia (222–224). Somatic variant data highlights the threshold effect associated with the RUNX1 activity, since reduced or absent activity associates with severity and prognosis of BMF and/or HM. The presence of somatic RUNX1 mutations in HM development, as well as the identification of RUNX1 as a germline cause of HM, highlights the possibility that other genes recurrently observed in sporadic HM may also contribute to inherited BMF/HM. Incorporating such data into existing guidelines for future variant stratification may be beneficial.

Epidemiologic studies have established that the incidence of leukemia may be influenced by a combination of germline abnormalities and environmental risk factors including lifestyle choices (i.e., smoking and obesity) (225), environmental exposures (i.e., cytotoxic agents and electrical power) (225, 226), medical history (i.e., regular aspirin use) (227), and other chemical and biological agents (i.e., radiation, retroviruses) (228). For example, the broad range of GATA2 deficiency syndrome phenotypes and penetrance implicate the involvement of environmental stressors that increase the likelihood of developing particular phenotypes (229). The role of environmental factors in clonal hematopoiesis emergence (e.g., metabolic syndromes causing chronic inflammation and chemotoxic exposure) may also be highly specific to the mutation that marks each stem cell clone (230). Despite these findings, and efforts to standardize variant interpretation and calling, environmental factors are currently neglected in TF/gene stratification. As knowledge of confounding environmental stressors become better known, a variant’s classification may differ for different individuals within a family, for instance, dependent on environmental influences such as is already done for responses to certain medications (231, 232).

## Predictive inclusions to BMF and/or HM predisposition TFs

Many TF networks have inherent redundancies that minimize the impact of disruption of any one TF to target genes’ expression levels. As such, phenotypes arise due to critical gene expression changes where a particular TF has a major role that drops expression or transcriptional activity below a critical threshold leading to disease. Importantly, this threshold may only be reached in some cases due to internal or external stresses and may be the reason why we see the somatic involvement of some gene variants in disease (for example sporadic HM), but not as pathogenic germline variants. Additionally, certain genetic variants that occur somatically in the bone marrow or blood may never be seen as germline because they are embryonic lethal. A framework utilizing in-silico pathogenic predictor scores, occurrence in oncogenic gene fusions, and previous disease association could provide insight into the oncogenic potential of novel TFs as BMF and/or HM predisposition genes. By highlighting them, others may include in their variant analyses, scrutiny, and curation approaches.

In this section, we will cover criteria that may aid in determining whether novel TFs may be disease causing. Highlighting this criterion, we will use TFs from our list of potential novel candidates (**Table 4**). We focus on two ETS TFs, which are part of the known transcriptional cluster model for hematopoiesis, ERG and PU.1 (36), and a ZF TF, GATA3. These three candidates all regulate central biological processes in hematopoiesis and have known oncogenic potential, which make them prime candidates as novel BMF and/or HM predisposition genes.

### Role in normal hematopoiesis

ERG, PU.1 and GATA3 are vital TFs in normal hematopoiesis, involved in the regulation of broad transcriptional programs in lineage differentiation of various types of mature blood cells. ERG has a unique and multifaceted role within the normal HSC compartment, as it is particularly critical for B lymphopoiesis, progenitor self-renewal and HSC function to sustain definitive hematopoiesis, often by preventing HSC exhaustion (83, 233–237). Despite this, hematopoietic specification and the initiation of definitive hematopoiesis is not ERG-dependent (236). PU.1 is another ‘pioneer’ TF, heavily reliant on its large transcriptional program and alternative mechanisms (SWI/SNF, and c-Jun) (234, 235) to assist hematopoiesis, particularly myeloid and B cell differentiation (238, 239). GATA family of TFs (GATA1-3) are vital determinants of multilineage hematopoiesis, and so far, germline variants in GATA1 and GATA2 have an established role in HM development, particularly GATA2 as one of the most characterized predisposition genes. Given the crucial role of the GATA TF family in both normal and leukemic hematopoiesis, it is imperative that much like its familial counterparts, germline mutations in GATA3 will likely also have pathogenic consequences. GATA3 is essential for the development, maintenance, survival and proliferation of early T-cell progenitors and HSC emergence, despite its expressional absence in most hematopoietic cells. (**Figure 2**).

### Disease phenotypes and malignancy associations

The central involvement of ERG, PU.1 and GATA3 in hematopoietic lineage differentiation and their embryonic lethality occurring concomitantly with the onset of definitive hematopoiesis makes them primal candidates for hematopoietic predisposition genes (240–242). Both ETS TFs, ERG and PU.1, have been linked to hematological-related phenotypes in humans, and all three TFs have exhibited hematologic-related phenotypes in mice, further strengthening their candidacy (**Table 4**). Somatic LOF mutations in all three TFs have been observed in cancer with GATA3 exhibiting the highest frequency of LOF mutations (over 700).

ERG is constitutively expressed and exerts multiple hematopoietic/non-hematopoietic homeostatic functions, though its pathological dysregulation outside of this homeostatic range has previously associated the protein with HM amongst other diseases. ERG’s overexpression has been identified as a biomarker correlated with adverse AML clinical outcomes, and its deregulation (i.e., intragenic deletions) found in ALL patients, has been the result of favorable outcomes (70, 96–100, 243, 244). This suggests that like GATA2 germline mutations, ERG may not uniformly disrupt expression and function in all cellular contexts. Traditional biological models (i.e., knock-out, knock-in and knockdown mouse models) have also implicated ERG in BMF and/or HM phenotypes. The characterization of a (mouse) germline ERG variant (p.S305P) residing in BMF (thrombocytopenia) also alludes to the possibility of ERG as a low penetrant leukemic predisposition gene (83, 233, 245). In other diseases, ERG-dependent transcription has been shown to modulate cardiovascular disease (246), and germline LOF ERG variants have been reported to lead to lymphedema (247), see also in ~10-15% of germline GATA2 cases (74). Perhaps the strongest example of oncogenic potential is ERG’s involvement in chromosomal translocations. Gene fusions between androgen-regulated genes and ERG (e.g., TMPRSS2-ERG) occur in ~50% of prostate cancers, with the alteration resulting in the presence of ERG overexpression in both early and late-stage prostate cancer (248, 249). This overexpression drives oncogenic effects such as increased cell growth, increased expression of neurotransmitter receptors and promotes tumor development, making the fusion a standard biomarker for diagnosis and stratification (249, 250). Hematopoietic-associated fusions involving ERG also extend to EWSR1-ERG in 5-10% of Ewing’s sarcoma, and ELF4-ERG and FUS-ERG in acute myeloid leukemia (96, 251, 252).

Despite its heavy-weighted importance in blood formation, large HM-related phenotypic link in mice, and embryonic lethality seen in homozygous mice, (**Table 4**) pathogenic germline SPI1 (encodes PU.1) variants are also yet to be found. The dysregulation of PU.1 and its consequent role in HM-related pathology, however, has been well studied. Specifically, PU.1 protein levels have been linked to the inhibition of cell division, cell cycle and leukemogenesis. PU.1 overexpression leads to differentiation blocks and thus acute erythroleukemia (101), but the reduction of PU.1 expression (and consequently gene network) has been shown to aid in leukemic transformation by many mechanisms including TET2 deficiency (253, 254), differentiation blocks and cellular expansion involving synergistic combination of PRC2 and HDAC1 (255), and at the post-transcriptional level, sustained expression of miR-155 (256). PU.1’s well established role in leukemogenesis makes it an attractive target for therapeutic intervention; for example, the inhibition of miR-155 to inhibit cell growth, controlled by dysregulated PU.1, has been proposed as a potential pathway to impact outcomes in HM (257).

Although PU.1 has a well-established role in leukemogenesis, the prevalence of somatic mutations remains relatively low. Only 0.32% of somatic point mutations in PU.1 are reported in hematopoietic and lymphoid tissues in the COSMIC database, (**Table 2**) and one recurrent somatic PU.1 mutation shown to be associated with a poor prognosis of Waldenstrom macroglobulinemia, underscoring the rarity of PU.1 somatic mutations. The variant (p.Q226E) has been shown to modify DNA binding specificity and transactivation capacity on ETS-like binding sites (258). *De novo* aberrations in PU.1 have also been linked to autosomal dominant “Agammaglobulinemia, including lymphopenia, neutropenia and impaired B-cell development” phenotypes. Patient cells show loss of PU.1 protein expression, which is consistent with a LOF and haploinsufficiency mechanism (259). These observations suggest a possibility of reduced penetrance and variable expressivity for HM-related phenotypes.

GATA3’s pivotal role in developmental T lymphopoiesis, particularly in late stages of T-cell differentiation (242), may assist in predicting mutational burden, although its role in the progression of lymphoid-related leukemias is largely unexplored. Dysregulated GATA3 expression has previously been implicated in a subgroup of T-ALL patients, where both high and low GATA3 expression resulted in changes of target gene expression clusters (260). This dysregulation was initially reported to have no significant changes in clinical outcomes (261); however, a subsequent study revealed that GATA3 dysregulation was associated with poorer survival and adverse prognostic implications (262). In biological models, GATA3 aberration has been shown to disrupt signaling cascades, one of which, a GATA3-dependent cytokine (i.e., IL-13), has been shown to promote the growth and survival of malignant T-cells (260). A GATA3 mutation in zebrafish embryos (p.R276Q) was also hypothesized to collectively affect T-cell proliferation and differentiation, eventually contributing to the pathogenesis of T-ALL, highlighting an emerging role for GATA3 in HM (263).

Dysregulation of GATA3 has also been associated with other diseases, including breast cancer, where reduced GATA3 expression was linked with a poorer prognosis and unfavorable tumor phenotype (264), and where mutations in exon 6 of GATA3 were detected in >50% of tested patients. Interestingly, the study suggested a difference in GATA3 mutation type may have different outcomes, where intronic germline mutations correlate to better prognosis whilst protein coding variants do not (265).

### Pathogenicity predictors

Scores like pLI predict the tolerance of a gene due to the sum of variants causing premature termination of a protein and are often used to prioritize candidate genes when analyzing genomic data (266). Both ERG and PU.1 are highly intolerant to LOF (0.96, 0.98, respectively, **Table 2**, **Figure 3**) as are their DNA binding ETS domains. This contrasts with other ETS TFs, such as FEV, which has a much lower pLI score (0.01, **Table 3**) despite having a similarly intolerant DNA binding ETS domain. Despite the rarity of somatic mutations and absence of germline mutations in BMF/HM in ERG and SPI1, nine pathogenic ClinVar variants (in FLI, ETV6 and ERF) all located within the ETS DNA binding domain, are directly aligned to corresponding amino acids in both PU.1 and ERG, which is highly suspicious of a similar disruptive effect on the associated protein’s function.

GATA3 also exhibits a high pLI pathogenicity predictor score of 0.9 (**Table 2**, **Figure 3**), and like its familial counterparts, has highly intolerant (ZF1) and intolerant (ZF2) DNA binding domains. Given the number of germline ClinVar reported pathogenic variants segregating in the highly conserved ZFs of both GATA1 and GATA2, it is unsurprising that over 20 variants are directly aligned to the same amino acid in GATA3. Interestingly, GATA3 has a reported gene-disease association to both deafness and renal dysplasia, phenotypes of which have also been reported in GATA2 deficiency syndrome (53, 267).

### Gene expression and interactions with known predisposition genes

Based on current reported BM and/or HM predisposition TF genes and correlation to their expression levels in various hematopoietic cell types, mRNA expression levels while informative are poorly predictive of whether variants in an expressed gene are likely to be pathogenic (**Figure 2**). This may be because of the difficulty in determining threshold levels of activity for each TF for normal function, and because mRNA levels don’t always correlate with protein or activity levels. It is also unsurprising because single cell studies show that despite the very low or absence of GATA2 expression in myeloid progenitor cells, the preferential loss of myeloid cells (e.g., monocytes) is a feature of GATA2 deficiency syndrome (268). The impact of both temporal and spatial expression of TFs is also important in the biology leading to genetic variants being pathogenic (i.e., detrimental in a way that promotes oncogenicity either under normal physiological conditions or stressed environments), along with interplay with cofactors and their expression profiles during hematopoietic processes.

The interconnectedness of the known TF BMF and/or HM predisposition gene network is demonstrated by reported protein-protein interactions (**Supplementary 1A**). Given phenotypic overlap and the biological pathways involved, it is likely novel genes would show a similar network of protein-protein interactions with this group of TFs. Using STRING analysis, it is clear that the majority of genes in our potential TF predisposition list show a strong interconnectedness with our known TF network list (**Supplementary 1**) (12). As an example, GATA3 shows strong links with several of the well-known predisposition TFs including RUNX1 and GATA2. Utilizing the network approach, FEV is an outlier of the network suggesting it is less likely to be an important TF in BMF/HM predisposition without known interactions with other TFs important to disease pathogenesis. The low pLI score and negligible number of somatic variants in COSMIC do not support a role for pathogenicity alongside the temporal expression of FEV during prenatal hematopoiesis (269).

ChIP Enrichment analysis (ChEA) has been utilized to analyze over 70 gene set libraries to identify consensus target genes for selected TFs (13). In cases where data for a particular TF was available from multiple experiments, a set intersection approach was employed to obtain a consensus on the top 9 interactions based on their p-value. (**Table 1**) Known BMF/HM TFs were among the top three interacting partners of 7 of our selected TFs (known and strong candidate BMF/HM predisposition genes), indicating that the network of genes associated with BMF/HM predisposition is highly interconnected. This also emphasizes the role of candidate inclusion TFs (GATA3, PU.1, ERG) in this gene network. Interestingly, ERG’s direct regulation of RUNX1 and GATA2, and its (dose-dependent) absence, has been shown to reduce the expression of RUNX1 and GATA2, suggesting a possible mechanism of oncogenicity for ERG LOF/partial LOF variants (236).

### Clonal hematopoiesis of indeterminate potential

**CHIP** is increasingly being investigated in hematological predisposition syndromes as a contributing factor in leukemogenesis. In the healthy population CHIP is a risk factor for HM, cardiac, and pulmonary disorders, and mortality (270), however this occurs primarily in the aging population (10% in >70 years). It is becoming apparent that early-onset CHIP occurs in TF predisposition HM syndromes including GATA2 and RUNX1 (271). What is not yet determined is whether this phenomenon of early-onset CHIP is largely attributed to TF HM predisposition genes or all HM predisposition genes. A recent study showing CHIP associated with ANKRD26 germline HM might argue against it being specific to TF predisposition genes, however this study was underpowered, warranting further investigation of larger patient numbers (95). Interestingly, germline ANKRD26 HM phenocopies RUNX1 FPD-MM, with pathogenic variants in ANKRD26 occurring in the 5’UTR and disrupting RUNX1 and FLI1 TF binding sites providing a link to altered TF biology and CHIP. [9,266] It might be anticipated that those predisposition genes, such as TFs and/or epigenetic regulators, that have an impact on a multitude of pathways during hematopoiesis are more likely to impact pathways predisposing to CHIP mutation accumulation such as DNA damage pathways, which has been implicated in RUNX1 FPD-MM. Interestingly, the majority of CHIP mutations are in genes involved in epigenetic regulation and mutations in TFs are rarely observed. The reason for this could be two-fold, firstly TF mutations, may lead to deregulation of HSC senescence and proliferation resulting in a downstream impact on cellular survival, or lead to activation of terminal differentiation of HSC which would not result in the clonal advantage required for CHIP, alternatively, the effect on gene-regulation networks and associated epigenetic changes results in progression to malignancy. An example of this is in RUNX1 FPD-MM, in unaffected individuals with germline RUNX1 variants we do not detect CHIP in RUNX1 however biallelic RUNX1 variants are the most common event associated with malignancy (229).

CHIP is associated with distinct DNA methylation (DNAm) patterns. Similarly, DNAm patterns change with both aging and in AML. CHIP has been postulated to act as a “molecular clock”, with the presence of CHIP associated with an increase in age as measured by DNAm-biomarkers of aging (272).Why germline variants in TFs such as RUNX1 and GATA2, might accelerate this molecular clock still remains to be understood. Aged HSCs, as well as those with forced increases in cellular divisions, were shown to lead to alterations in DNAm (273).CHIP is likely reflecting the role of these TFs in regulating cellular processes such as mitosis, cell divisions, senescence, DNA damage and inflammatory responses, which can all lead to changes in the epigenome (274). Interestingly, the most common CHIP genes include DNMT3A and TET2, both of which are regulators of DNAm (275). It has been shown that both DNMT3A- and TET2-CHIP associated CpG DNAm patterns are enriched for ERG binding sites, while DNMT3A-CHIP sites are enriched for RUNX1 and RUNX2, GATA TF subfamily binding sites and TET2-CHIP sites enriched for ETS transcription factors (57). This is not unexpected given these are all TFs involved in hematopoiesis and implicated in leukemogenesis, thus providing a link between CHIP, DNAm changes and downstream consequences of altered HSC self-renewal and risk of leukemia development possibly mediated by these TFs.

Identification of CHIP prior to malignancy development holds great potential for future development of preventative therapies in TF HM predisposition genes. This may be through small molecule inhibitors designed to target specific epigenetic regulators, such as TET inhibitors (21) or designing drugs to target CHIP regulated pathways. An elegant study in zebrafish, demonstrated clonal dominance of HSPCs associated with loss-of-function variants in common CHIP genes asxl1,dnmt8(DNMT3A ortholog), and tp53 which was also associated with a pro-inflammatory environment to which these clones are resistant (22). This provides an opportunity to target inflammatory signaling pathways to prevent clonal outgrowth of HSPCs, such as through targeting inflammatory signaling mediated by NR4A1 which has been shown to provide clonal dominance (22).

### Gene ontology

**Figure 4:**
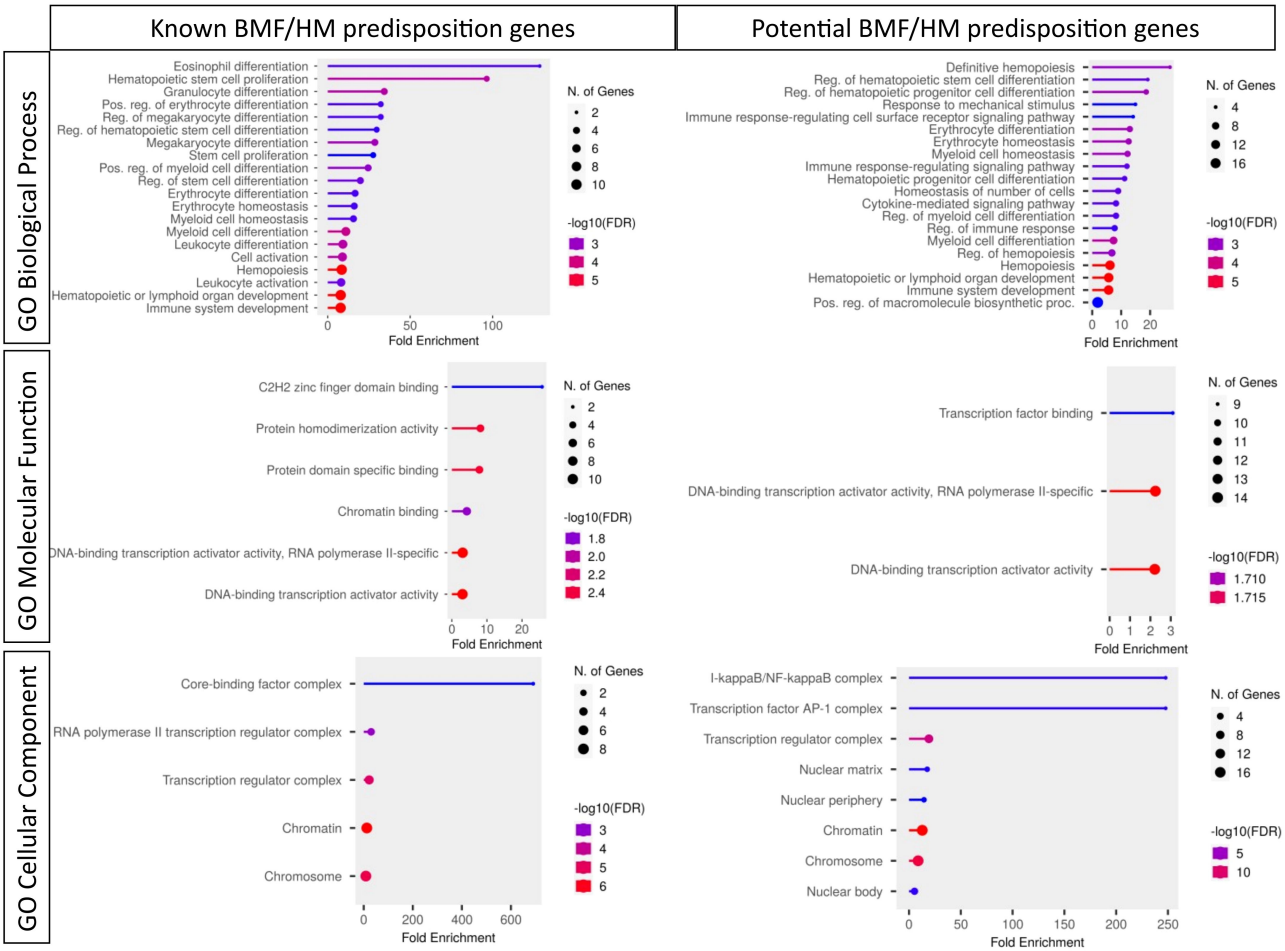
Gene Ontology of TFs implicated in hematological development and disease. Using ShinyGO0.77 analysis software (31), the known BMF/HM predisposition list in **Table 1** and the potential list of BMF/HM predisposition genes in **Table 4** (including *ERG*, *GATA3* and *SPI1*) were analyzed for enrichment of GO terms in biological process and Molecular functions using all known human TF genes as background (i.e., normalization). All protein coding genes were used for background in GO cellular component analysis as there was no enrichment when using TFs as background.

Gene ontology (GO) resources have long been used to characterize and identify enrichment of biological processes, molecular functions, and cellular components relevant to particular gene sets and different disease states. Ontology terms and databases can provide a baseline to organize, standardize and prioritize TFs using different biological categories important to BMF and HM development. This is evidenced when searching for GO terms enriched in our BMF and/or HM predisposition gene list (**Table 1**). There is a significant enrichment of biological process pertaining to hematopoiesis including stem cell proliferation and differentiation, and in particular differentiation to myeloid cellular lineages all of which are important processes for the development of BMF and HM, and the bias towards myeloid malignancies often observed with these genes (**Table 1**, **Figure 4**). GO analysis of the list of potential BMF/HM TF predisposition genes, closely correlates with our known predisposition gene list including enrichment of hematopoietic differentiation and stem cell regulation (**Table 3**, **Figure 4**). Interestingly, significant enrichment of immune response pathways and cytokine signaling are also observed. These pathways are impacted by external stimuli and stressors as well as aging which could account for why these genes have not yet been discovered as predisposing, as it is likely these factors could act as modifiers having an impact on penetrance and clinical presentation, complicating the identification of more penetrant causative genes.

The absence of germline variation in some essential hematological TFs, could imply that a defective gene product would lead to a severe disruption in blood cell differentiation and a condition incompatible with life. This is supported by the paucity of genetic variation in the normal population (**Table 2**) and reported cases of miscarriages in families with pathogenic variants in multiple predisposition genes including *RUNX1*, *GATA2* and *MECOM* (**Table 2**) (53, 129). Furthermore, due to the role of these TFs in other organ systems, with genetic alterations leading to severe disorders (like *GATA3* mutations leading to HDR syndrome) (276), it reduces the possibility of being discovery in the context of HM predisposition. Studies focusing on stillbirth and fetal lethal conditions, detected a single case of a disease-causing variant in a novel hematopoietic TF gene (*GATA3*), terminated at gestational week 20 (**Table 2**) (277, 278). The limited number of associations with fetal death could be attributed to timing of genetic testing. First blood cells differentiate from extraembryonic mesoderm around the 7th day of embryonic development, and early miscarriages are often not genetically tested. The presence of truncating variants in COSMIC could suggest that LOF variants in some TF genes are intolerant to germline variation and may only be acquired, where the temporal and spatial origin of the expressed variant determining the site of malignant tissue (29, 73) Conversely, low penetrant variants in genes tolerant to variation are often not recognized as their individual contribution to disease is limited. However, they may contribute in combination with other genetic variants and/or under influence by environmental factors. Only with global data-sharing approaches and continued expansion of availability to routine genomic sequencing will there be the possibility of reaching the statistical power required to discover risk alleles for BMF and/or HM predisposition.

## Clinical implications

Improving understanding of the genetic landscape of predisposition to BMF and/or HM is imperative to provide advice on clinical management and monitoring regimes, tailored therapies, and design targeted therapies (9). While the availability of carefully curated gene panels has led to improving the diagnostic yield of germline causes of BMF and HM, the use of such panels has caveats as novel genes such as those in the potential list of predisposition TF (**Table 3**) will be missed. As the cost of NGS technologies continues to decline and the access to these technologies’ increases, it is inevitable that more predisposing mutations in novel TFs involved in hematopoietic pathways will be added to the list of known predisposing genes. It can be challenging for laboratories and/or hematologists not experienced in germline evaluation to identify individuals most suitable for extensive evaluation for a germline cause (270). A familial history is not always observed, confounded by phenotypic heterogeneity and highly variable penetrance associated with different types of predisposing variants in TFs which will undoubtedly impact multiple different cellular and differentiation pathways, and be impacted by common polygenic modifier variants and external stressors. To alleviate some of these difficulties, laboratories are turning to artificial intelligence machine learning technologies to stratify clinical and genetic variables, to better classify inherited versus sporadic causes of BMF/HM, triaging those individuals who are most likely to benefit from more extensive genetic investigation (271). The importance of identifying a germline cause of disease cannot be understated, with implications for surveillance, treatment protocols and clinical care. In a large study, identification of a germline cause of hematopoietic predisposition syndromes impacted the clinical management of 91% of participants (279). For example, GATA2 carriers considered for BMT/HSCT often require specific protocols including high-dose post-transplant cyclophosphamide after transplant to avoid graft-versus host disease (272). Additionally, due to the likelihood of requirement for HSCT in germline GATA2 carriers, prophylactic HSCT before transformation may be considered (273). Whereas, HSCT is often not recommended for germline *CEBPA* patients, due to good responses to chemotherapy, and the risk of morbidity and mortality associated with HSCT. However, continued molecular monitoring is recommended due to novel somatic variants identified at relapse (67). HSCT transplant is often considered as the only curative therapy for HM, with matched-related donors often considered as the optimal choice to reduce the risk of transplant rejection and graft versus host disease. Knowledge of a germline causal variant provides the means to screen potential donors thereby reducing the risk of donor-derived leukemias (274). Continued molecular monitoring of carriers is crucial, and evidence is increasing of the unique suite of somatic mutations most likely to occur for each TF predisposition gene during disease progression. As gene/mutation specific drugs become more common-place, clinical management, treatment, and prognosis may change for affected carrier individuals. Clinically, it will be important to consider that these TF BMF/HM predisposition disorders may not be purely monogenic disorders. It is likely that multiple genetic variants (rare and common) in different genes of these highly regulated TF pathways during hematopoiesis are contributing to variable penetrance and phenotypes. For example, a germline RUNX1 family was subsequently found to carry pathogenic germline variants in DDX41 and ANKRD26 (both associated with cytopenia’s and HM) in different branches of the family, and several individuals carried multiple variants (9). Future diagnosis/prognosis, management and possible therapy will likely include the use of polygenic risk scores accounting for the pathogenicity or predisposing capacity of the sum of germline variants on a case-by-case rather than familial basis.

## Conclusion

TFs play a key role in normal hematopoiesis that when perturbed can give rise to a range of phenotypes including BMF and/or HM. As genomic testing becomes more widely adopted and more comprehensive in its nature, it is likely that germline predisposing variants will be identified in novel genes and non-coding regions of known hematopoietic TF genes. It is likely that these will provide different “fertile soil”, predisposing to subtly new collections of phenotypes with unique penetrance’s under different stressors leading to BMF, cytopenia’s and/or clonal outgrowth as HM.

## Author contributions

JZ, CCH, PA, AB, HS and CNH contributed to the design, ideas and structure of the manuscript. JZ, CCH and CNH wrote the review. All authors contributed to the article and approved the submitted version.

## Supplementary material

Supplementary Figure 1 **(A)** Known interacting partners of HM and/or BMF predisposition TFs. The STRING database was used to map the proteins which are part of a physical complex with each known TF. In the bold blue text are the known predisposition TFs. Line thickness correlates with the strength of the association. **(B)** Interactions between TFs implicated in predisposition to BMF and HM. The STRING database was used to map known and predicted protein-protein interactions (12). Interactions include direct (physical) and indirect (functional) associations between, (i) known predisposition TFs and (ii) known and predicted predisposition TFs. Line thickness correlates with the strength of the association.
