# Supplementary figures and images for: Transcription factor genetics and biology in predisposition to bone marrow failure and hematological malignancy
Source: Front Oncol. 2023 Jun 12;13:1183318. doi: 10.3389/fonc.2023.1183318 (PMC10291195; doi:10.3389/fonc.2023.1183318)

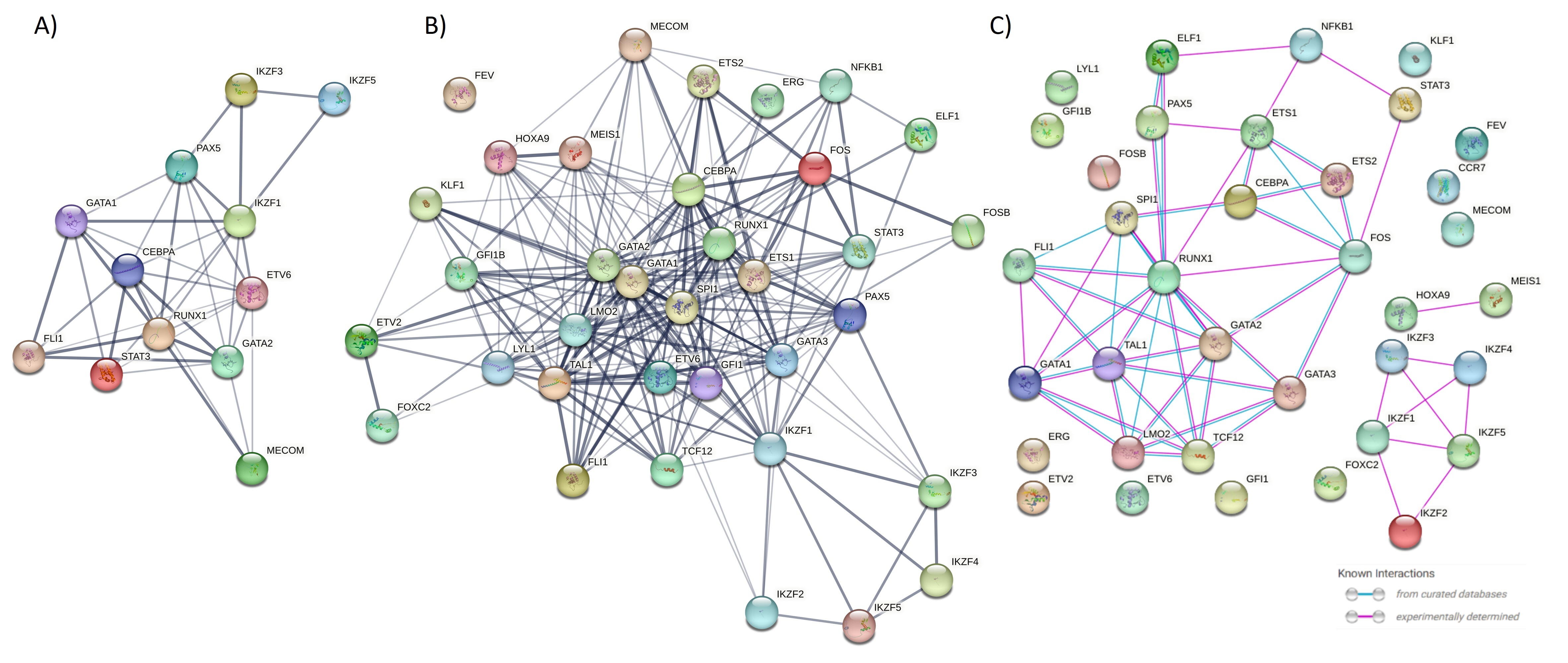

Supplement: Supplementary Figure 1 — (A) Known interacting partners of HM and/or BMF predisposition TFs. The STRING database was used to map the proteins which are part of a physical complex with each known TF. In the bold blue text are the known predisposition TFs. Line thickness correlates with the strength of the association. (B) Interactions between TFs implicated in predisposition to BMF and HM. The STRING database was used to map known and predicted protein-protein interactions (12). Interactions include direct (physical) and indirect (functional) associations between, (i) known predisposition TFs and (ii) known and predicted predisposition TFs. Line thickness correlates with the strength of the association. [file Image_1.jpeg]
